# Human migration and the spread of malaria parasites to the New World

**DOI:** 10.1038/s41598-018-19554-0

**Published:** 2018-01-31

**Authors:** Priscila T. Rodrigues, Hugo O. Valdivia, Thais C. de Oliveira, João Marcelo P. Alves, Ana Maria R. C. Duarte, Crispim Cerutti-Junior, Julyana C. Buery, Cristiana F. A. Brito, Júlio César de Souza, Zelinda M. B. Hirano, Marina G. Bueno, José Luiz Catão-Dias, Rosely S. Malafronte, Simone Ladeia-Andrade, Toshihiro Mita, Ana Maria Santamaria, José E. Calzada, Indah S. Tantular, Fumihiko Kawamoto, Leonie R. J. Raijmakers, Ivo Mueller, M. Andreina Pacheco, Ananias A. Escalante, Ingrid Felger, Marcelo U. Ferreira

**Affiliations:** 1 Department of Parasitology, Institute of Biomedical Sciences, University of São Paulo, 05508-900 São Paulo, Brazil; 2 Laboratory of Immunology and Parasite Genomics, Institute of Biological Sciences, Federal University of Minas Gerais, Belo Horizonte, Brazil; 3 Laboratory of Biochemistry and Molecular Biology, Superintendency for the Control of Endemics (SUCEN), State Secretary of Health, São Paulo, Brazil; 4 Department of Social Medicine, Federal University of Espírito Santo, Vitória, Brazil; 5 Laboratory of Malaria, René Rachou Research Center, Oswaldo Cruz Foundation, Belo Horizonte, Brazil; 6 Regional University of Blumenau, Blumenau, Blumenau, Brazil; 7 Center of Biological Research of Indaial, Indaial, Brazil; 8 Department of Pathology, School of Veterinary Medicine and Animal Sciences, University of São Paulo, São Paulo, Brazil; 9 Laboratory of Protozoology, Institute of Tropical Medicine of São Paulo, University of São Paulo, São Paulo, Brazil; 10 Laboratory of Parasitic Diseases, Oswaldo Cruz Institute, Oswaldo Cruz Foundation, Rio de Janeiro, Brazil; 11 Department of Tropical Medicine and Parasitology, Juntendo University School of Medicine, Tokyo, Japan; 12 Department of Parasitology, Gorgas Memorial Institute of Health, Panama City, Panama; 13 Department of Parasitology, Faculty of Medicine, and Institute of Tropical Disease, Airlangga University, Surabaya, Indonesia; 14 Department of Social and Environmental Medicine, Institute of Scientific Research, Oita University, Oita, Japan; 15 Research Laboratory for Archaeology and the History of Art, University of Oxford, Oxford, United Kingdom; 16 Division of Population Health and Immunity, Walter and Eliza Hall Institute, Parkville, Victoria Australia; 17 Department of Parasites and Insect Vectors, Institut Pasteur, Paris, France; 18 Department of Biology, Institute for Genomics and Evolutionary Medicine, Temple University, Philadelphia, Pennsylvania United States of America; 19 Swiss Tropical and Public Health Institute, Basel, Switzerland; 20 University of Basel, Basel, Switzerland; 21 Present Address: U.S. Naval Medical Research Unit No. 6, Bellavista, Callao Peru

## Abstract

We examined the mitogenomes of a large global collection of human malaria parasites to explore how and when *Plasmodium falciparum* and *P. vivax* entered the Americas. We found evidence of a significant contribution of African and South Asian lineages to present-day New World malaria parasites with additional *P. vivax* lineages appearing to originate from Melanesia that were putatively carried by the Australasian peoples who contributed genes to Native Americans. Importantly, mitochondrial lineages of the *P. vivax*-like species *P. simium* are shared by platyrrhine monkeys and humans in the Atlantic Forest ecosystem, but not across the Amazon, which most likely resulted from one or a few recent human-to-monkey transfers. While enslaved Africans were likely the main carriers of *P. falciparum* mitochondrial lineages into the Americas after the conquest, additional parasites carried by Australasian peoples in pre-Columbian times may have contributed to the extensive diversity of extant local populations of *P. vivax*.

## Introduction

The Americas were the last continent to be settled by modern humans. Approximately 15,000 years ago, the first Americans crossed the land bridge that connected Siberia to Alaska in the late Pleistocene. The precise date of the earliest arrival, number of founding events, and precise geographic source of peoples who migrated to the New World remain uncertain^1^.

These early migrants are unlikely to have carried malaria parasites through the cold and arid route to Alaska^2,3^. Accordingly, present-day Native Amerindians do not show genetic traits conferring protection from malaria infection or severity, such as hemoglobinopathies, sickle-cell trait, glucose-6-phosphate dehydrogenase (G6PD) deficiency, and Duffy antigen/receptor for chemokines (DARC) negativity, which have been selected in African and Eurasian populations heavily exposed to malaria^4^. Moreover, reports of conquerors and early settlers fail to mention severe malaria-like illnesses in indigenous populations soon after European contact^5–7^. Therefore, the most virulent human malaria parasite, *Plasmodium falciparum*, most likely entered the New World after European contact and was carried by Africans brought to the Americas between the mid-1500s and mid-1800s^8^ and settlers from the main colonizing nations, Portugal and Spain, where malaria was endemic at the time of conquest^9,10^.

How and when the less deadly species *P. vivax* arrived in the New World remains controversial. This parasite was endemic to southern Europe until the mid-1900s^9,10^, but it was rare in West and Central Africa, the origin of most enslaved peoples displaced to the Americas. Nearly all of them lack DARC, a key receptor for red blood cell invasion by *P. vivax*, and are therefore virtually resistant to blood-stage infection with this species^11^. Parasites may also have entered the New World in pre- and post-Columbian times with migrants from the Asian mainland and the Western Pacific^3,12,13^, further contributing to the surprisingly high genetic diversity of *P. vivax* in the Americas^14–17^. Archaeological evidence for infection with *P. vivax* in pre-Columbian Native Americans is currently limited to a single report of species-specific antigens visualized by immunohistochemistry in South American mummies dating from 3,000 to 600 years ago^18^, but confirmation of *P. vivax* infection with more specific molecular techniques is still required^19^. Importantly, specific antibodies failed to detect *P. falciparum* antigens in these same samples^18^.

Here, we analyze a large global sample of mitochondrial genomes (mitogenomes) to test competing hypotheses about the geographic origins of New World human malaria parasites^2,7,13,20–22^. We search for signatures of ancestral source populations in isolates of *P. falciparum* and *P. vivax* currently circulating in the Americas and of a *P. vivax*-like species, *P. simium*, which infects platyrrhine monkeys of the Atlantic Forest of South and Southeast Brazil. We find significant gene flow from Africa and South Asia to New World populations of malaria parasites with some additional genetic contribution of Melanesian lineages to local *P. vivax* strains. Because of the low diversity of *P. simium* lineages circulating in monkeys and humans in the Atlantic Forest ecosystem compared with *P. vivax* strains from across the country, we argue for a recent human-to-monkey transfer of these *P. vivax*-like parasites.

## Results

### Global and regional diversity of malaria parasite mitogenomes

Overall, we found less mitochondrial DNA diversity in *P. falciparum* than in *P. vivax* populations worldwide (Table 1; Supplementary Tables 1 and 2). We identified 330 single-nucleotide polymorphisms (SNPs) and 325 unique haplotypes in 1,795 mitogenomes from six regional *P. falciparum* populations, namely, populations in Africa (AFR), South America (SAM), Central America (CAM), South Asia (SOA), Southeast Asia (SEA), and Melanesia (MEL). The 941 mitogenomes from *P. vivax* populations in AFR, SAM, CAM (including Mexico), Middle East and Central Asia combined (MCA), SOA, SEA, East Asia (EAS), and MEL comprised 348 SNPs and 405 unique haplotypes.

**Table 1:** Global and regional levels of genetic diversity in *Plasmodium falciparum* and *P. vivax* mitogenomes.

Regional population	No. *P. falciparum* Isolates	Nucleotide diversity		*H*^d^ (SD)	No. *P. vivax* isolates	Nucleotide diversity		*H*^d^ (SD)^b^
		π^a^ (SD)^b^	θ*s*^c^ (SD)^b^			π^a^ (SD)^b^	θ*s*^c^ (SD)^b^	
AFR^e^	629	0.00026 (0.00001)	0.00486 (0.00094)	0.775 (0.014)	90	0.00043 (0.00005)	0.00254 (0.00068)	0.7930 (0.047)
CAM^f^	8	0.00035 (0.00007)	0.00027 (0.00017)	0.857 (0.108)	68	0.00008 (0.00002)	0.00040 (0.00015)	0.3510 (0.076)
EAS^g^	—	—	—	—	125	0.00082 (0.00005)	0.00121 (0.00034)	0.9170 (0.015)
MCA^h^	—	—	—	—	30	0.00060 (0.00008)	0.00126 (0.00044)	0.9490 (0.023)
MEL^i^	308	0.00028 (0.00002)	0.00113 (0.00028)	0.740 (0.019)	128	0.00045 (0.00005)	0.00178 (0.00047)	0.8669 (0.023)
SAM^j^	237	0.00018 (0.00002)	0.00160 (0.00039)	0.551(0.039)	238	0.00058 (0.00030)	0.00330 (0.00075)	0.9291 (0.012)
SEA^k^	532	0.00028 (0.00001)	0.00139 (0.00031)	0.773 (0.015)	160	0.00087 (0.00003)	0.00280 (0.00068)	0.9700 (0.006)
SOA^l^	81	0.00036 (0.00004)	0.00094 (0.00029)	0.858 (0.027)	102	0.00055 (0.00005)	0.00255 (0.00067)	0.9440 (0.018)
**Total**	**1795**	**0.00036 (0.00002)**	**0.00708 (0.00118)**	**0.887 (0.0047)**	**941**	**0.00085** (**0.00002)**	**0.00807** (**0.00014)**	**0.9732** (**0.0027)**

^a^π = average number of pairwise nucleotide differences per site; ^b^SD = standard deviation; ^c^θ_*S*_ = standardized number of segregating sites; ^d^*H = *haplotype diversity; ^e^AFR = Africa; ^f^CAM = Central America; ^g^EAS = East Asia (only *P. vivax*); ^h^MCA = Middle East and Central Asia (only *P. vivax*); ^i^MEL = Melanesia; ^j^SAM = South America; ^k^SEA = Southeast Asia; and ^l^SOA = South Asia.

### Geographic subdivisions in worldwide malaria parasite populations

**Figure 1:**
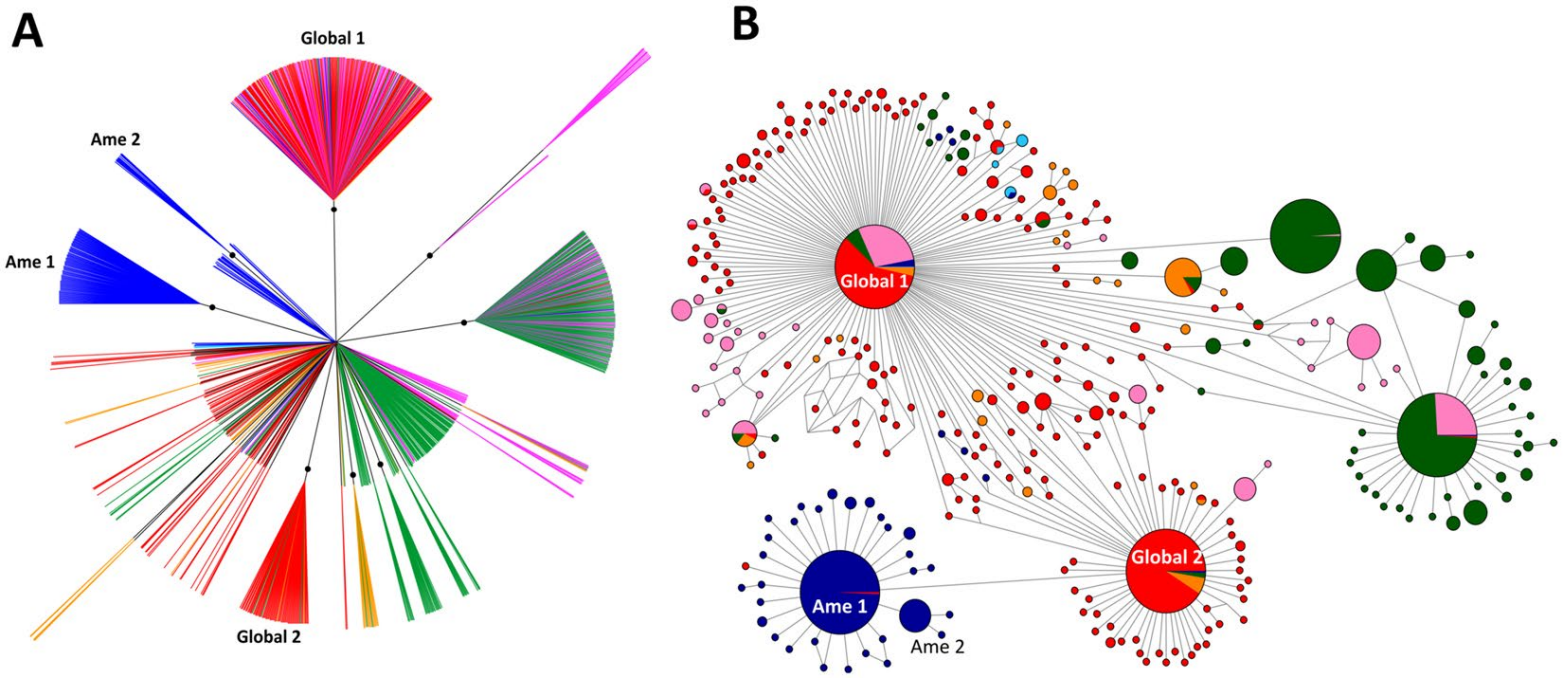
Bayesian phylogenetic tree (**A**) and median-joining network (**B**) of the global sample of *Plasmodium falciparum* mitochondrial lineages (n = 1795). Circle sizes in B are proportional to haplotype frequencies, and pairs of haplotypes connected by a straight line differ by a single mutational step. The following color code was used to identify the geographic origin of parasites: red = Africa, dark blue = South America, light blue = Central America, orange = South Asia, green = Southeast Asia, and pink = Melanesia. Branches with posterior probabilities >0.70 are indicated by black circles in the phylogenetic tree; selected well-supported clades indicated in the figure (Ame1, Ame2, Global1, and Global2) are further discussed in the main text.

Bayesian phylogenetic analysis revealed a clear geographic structure in the global *P. falciparum* population (Fig. 1A), which is consistent with independent regional colonization events^23,24^. The vast majority (81.0%) of SAM samples (n = 237) cluster in one of three well-supported clades (posterior probability > 0.7), each comprising a single haplotype. Two of these clades, Ame1 (157 samples from SAM [Brazil, Venezuela, Peru, and Ecuador] and 1 from AFR) and Ame2 (22 samples from SAM [all from Brazil]), are closely related to each other, as indicated by their single-step connection in the median-joining network shown in Fig. 1B. The third clade comprising SAM samples, Global1, is the largest one in the phylogeny with 450 samples from all regions (263 from AFR, 129 from MEL, 29 from SEA, 15 from SOA, 13 from SAM [all from Brazil], and 1 from CAM). Interestingly, the partial mitochondrial genome retrieved from an old European sample of *P. falciparum*^25^ is two mutational steps away from the *Global1* haplotype; it is shared by three SOA isolates, but not SAM samples (Fig. 3 of Gelabert and colleagues^25^). Most (57.8%) African lineages have the *Global1* and *Global2* haplotypes with the latter comprising 146 samples (133 from AFR, 9 from SOA, 3 from SEA, and 1 from SAM [Brazil]). Haplotypes *Global1* and *Global2* have a single-step connection to each other, whereas the most common South American haplotype, *Ame1*, is only one mutational step away from *Global2* and two steps away from *Global1* (Fig. 1B). SAM shares 5 haplotypes with other populations, and 4 of them with AFR (Supplementary Table 3). The relative genetic proximity between SAM and AFR mitochondrial lineages compared with those from other regions suggests that AFR is a major source of extant South American populations of *P. falciparum*. The origin of CAM lineages cannot be inferred from our phylogeny because 7 of the 8 CAM mitogenomes are placed in the undifferentiated cluster in the center of the tree (Fig. 1A).

Estimates of the Wright’s fixation index (*F*_ST_), a measure of divergence between populations due to genetic structure, revealed substantial genetic differentiation across regional populations of *P. falciparum*. All *F*_ST_ values were significantly different from zero, and 11 of the 15 pairwise comparisons yielded *F*_ST_ values > 0.15, which is consistent with relatively little gene flow between populations. SAM is the most divergent *P. falciparum* population with *F*_ST_ = 0.468 in the comparison with AFR and *F*_ST_ > 0.50 in all other comparisons with regional populations (Supplementary Table 4). *P. falciparum* mitochondrial lineages from AFR, however, show relatively little differentiation from SOA (*F*_ST_ = 0.091) and MEL (*F*_ST_ = 0.130).

**Figure 2:**
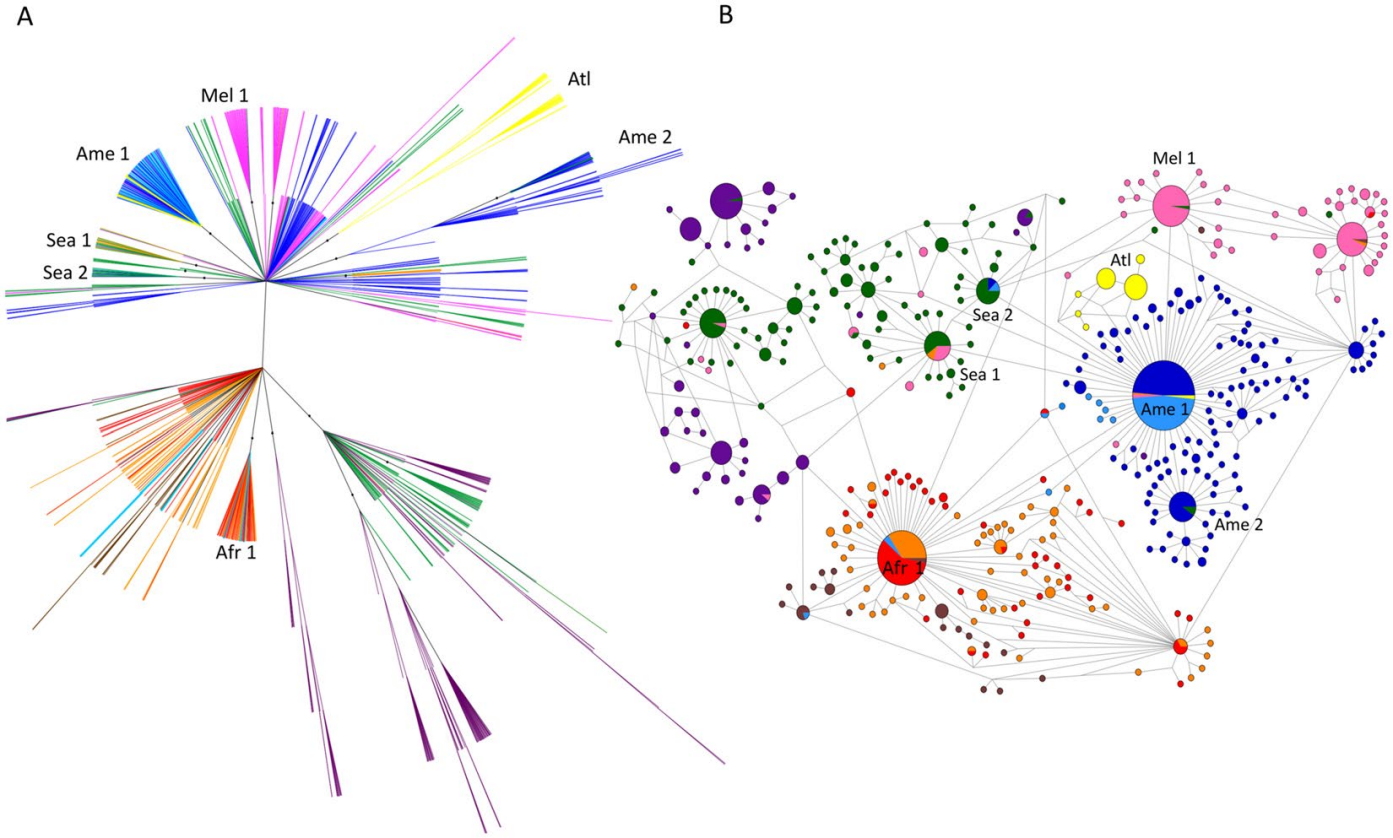
Bayesian phylogenetic tree (**A**) and median-joining network (**B**) of the global sample of *Plasmodium vivax* mitochondrial lineages (n = 941). Circle sizes in B are proportional to haplotype frequencies, and pairs of haplotypes connected by a straight line differ by a single mutational step. The following color code was used to identify the geographic origin of parasites: red = Africa, dark blue = South America, light blue = Central America and Mexico, yellow = Atlantic Forest from southeast and South Brazil, brown = Middle East and Central Asia, orange = South Asia, green = Southeast Asia, dark purple = East Asia, and pink = Melanesia. Branches with posterior probabilities >0.70 are indicated with black circles in the phylogenetic tree; selected well-supported clades indicated in the figure (Ame1, Ame2, Atl, Afr1, Mel1, Sea1, and Sea2) are further discussed in the main text.

South American mitochondrial lineages of *P. vivax* are widely spread across the Bayesian phylogenetic tree (Fig. 2A). Three well-supported clades (posterior probability > 0.7) comprise 47.5% of the 238 SAM samples: Ame1 (58 identical haplotypes from SAM, 54 from CAM, 1 from SOA, and 2 from MEL), Ame2 (25 lineages from SAM and 2 from SEA), and Atl (30 lineages from the Atlantic Forest of Brazil). Most remaining SAM lineages are placed in the central, star-shaped component of the tree. In contrast, the vast majority (85.3%) of the 68 CAM samples cluster in a single clade, Ame1. Three additional CAM samples (but none from SAM) have the *Afr1* haplotype, which is shared by AFR (n = 40), SOA (n = 25), and MCA (n = 2) samples (Fig. 2B). Only 3 (2.5%) of the 119 haplotypes circulating in SAM are shared with other regional populations (CAM, SOA, SEA, and MEL), and none of them are shared with AFR (Supplementary Table 5).

The only currently available, partially sequenced *P. vivax* mitochondrial lineage from Europe is very close to *Ame1* (Fig. 2 of Gelabert and colleagues^25^). Interestingly, the *Ame1* haplotype occupies a central position in the median-joining network representing New World *P. vivax* lineages (Supplementary Fig. 1). Indeed, *Ame1* has single-step connections with haplotypes *Afr1* (shared by 44.5% of AFR and 24.5% of SOA samples and a few CAM and MCA lineages), *Mel1* (shared by 30.5% of MEL samples), *Sea1* (shared by SEA, SOA, and MEL samples), and *Sea2* (shared mostly with SEA but also with CAM and SAM samples [two of each]; Fig. 2B). *Ame1* has been hypothesized to be of European origin, but it is directly related to the original (putatively now extinct) African population, and it is ancestral to regional lineages, such as *Mel1*, *Sea1*, and *Sea2*^26^. Interestingly, nine mitochondrial lineages of *P. vivax-*like parasites previously recovered from great apes in Central Africa^27^ did not cluster together with human parasites from this continent. Instead, they are only 2–4 mutational steps away from the *Ame1* haplotype circulating in the New World (Supplementary Fig. 3), which is again consistent with the notion that present-day New World lineages are most closely related to original African parasites^26^.

Wright’s *F*_ST_ values > 0.15 were found in 24 of the 28 pairwise comparisons of regional *P. vivax* populations (Supplementary Table 6). Relatively little (although significant) genetic differentiation can be found between AFR and SOA (*F*_ST_ = 0.014), AFR and MCA (*F*_ST_ = 0.087), and SOA and MCA (*F*_ST_ = 0.075), which is consistent with the hypothesis of recolonization of AFR following the near fixation of DARC negativity in local human populations by *P. vivax* stocks from the Middle East and SOA^3,26^.

Interestingly, gene flow patterns across continents appear to differ markedly according to parasite species (Supplementary Tables 4 and 6). For example, the MEL population of *P. vivax* is less divergent from SAM (*F*_ST_ = 0.242) than it is from AFR, SOA, or SEA (*F*_ST_ between 0.320 and 0.406); in contrast, the MEL population of *P. falciparum* is very divergent from SAM (*F*_ST_ = 0.558), but not as divergent from AFR, SOA, or SEA (*F*_ST_ between 0.097 and 0.130). Therefore, *F*_ST_ estimates provide some evidence of gene flow between MEL and SAM populations of *P. vivax*–a finding to be further explored in subsequent analyses–but very little gene flow between the corresponding populations of *P. falciparum*.

**Figure 3:**
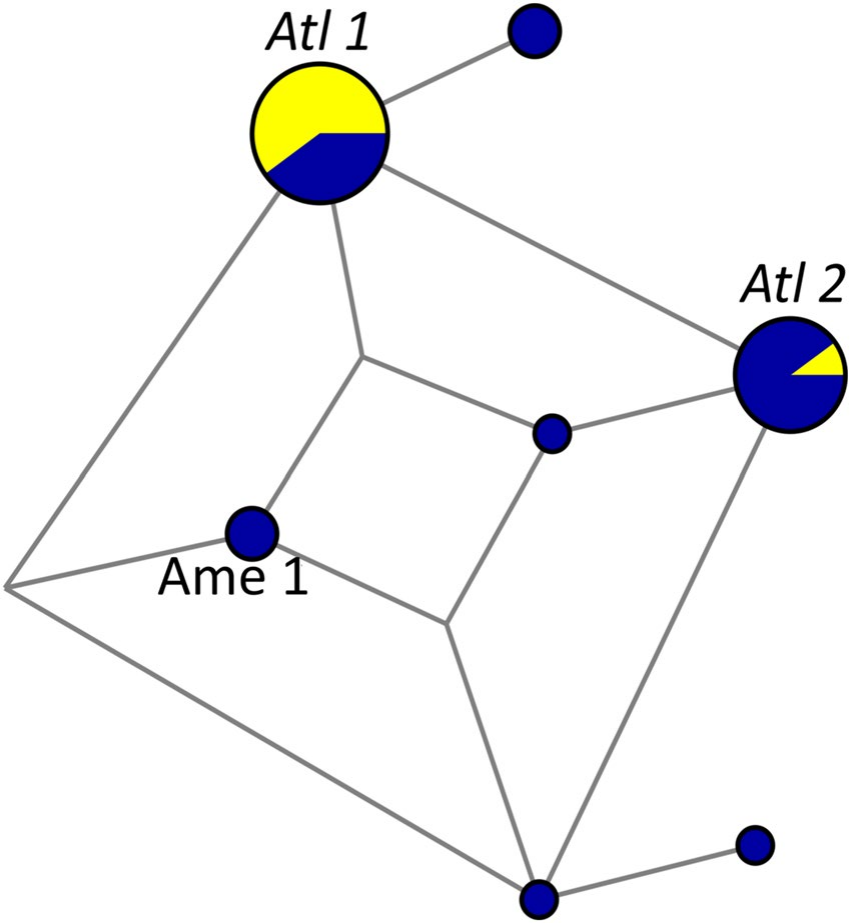
Median-joining network of *Plasmodium vivax/P. simium* mitochondrial lineages from the Atlantic Forest of Southeast and South Brazil. Circle sizes are proportional to haplotype frequencies, and pairs of haplotypes connected by a straight line differ by a single mutational step. Yellow indicates samples from monkeys, and blue indicates samples from humans. Haplotypes *Atl1*, *Atl2*, and *Ame1* are indicated. Sample collection sites and dates, as well as their respective hosts, are listed in Supplementary Table 7; sample collection sites are plotted on a map in Fig. S2.

Thirty-two *P. vivax*/*P. simium* lineages from humans and platyrrhine monkeys from forest-covered areas along the Atlantic Coast of Brazil have been included in our phylogenetic analysis. These ATL samples (represented in yellow in Fig. 2A,B; see also Fig. 3) originate from sites >2,000 km away from the Amazon Basin (Supplementary Fig. 2), the main *P. vivax*-endemic area in this country. They comprise *P. simium* isolates collected within a radius of 440 km from nine brown howler monkeys (*Alouatta clamitans*) and one black-fronted titi monkey (*Callicebus nigrifrons*) and *P. vivax* samples from 22 human infections within a radius of 40 km in the southeastern state of Espírito Santo (Supplementary Table 7). Regardless of their host, 30 of 32 ATL samples cluster in clade Atl (which contains two main haplotypes, *Atl1* and *Atl2*), whereas two human samples from Espírito Santo have the *Ame1* haplotype (Fig. 2A,B). Importantly, the time to the most recent common ancestor (TMRCA) of the entire Atl clade, which is estimated at 23,440 years before present (95% highest probability density [HPD] interval, 16,164–29,879 years before present), precedes the most likely dates of *P. vivax* arrival in the Americas. Nine of 10 *P. simium* samples share the *Atl1* haplotype, which is also found in 5 *P. vivax* samples recovered from humans living 700-1,500 km north of the *P. simium* collection sites (Fig. 3 and Supplementary Table 7). The *Atl2* haplotype, which was recovered from the original Fonseca MRA-353 isolate collected in the mid-1960s in São Paulo, is shared by 9 human *P. vivax* samples collected in Espírito Santo (over 700 km north of São Paulo) in the early 2000s (Supplementary Table 7) and by three patients from Rio de Janeiro^28^. The two human samples from the Atlantic Forest with the *Ame1* haplotype were most likely imported from the Amazon Basin. Importantly, however, the *Atl1* and *Atl2* lineages that circulate in humans and platyrrhine monkeys in south and southeast Brazil–and differ from each other by the A3325T nucleotide substitution at the *cox1* locus–have no clear connection with *P. vivax* populations from other continents (Fig. 2A,B). Two private SNPs mapping to the *cox1* gene (T4134C and A4468G) show potential for distinguishing autochthonous and potentially zoonotic *P. vivax* infections from the Atlantic Forest of Brazil from those imported from the Amazon Basin or elsewhere (Supplementary Table 8).

### Demographic history and divergence time

Several findings suggest a past demographic expansion of human malaria parasites worldwide. First, both Tajima’s and Fu’s tests yielded significantly negative values for most regional populations of *P. falciparum*, except for CAM and SOA (for the latter, only Tajima’s *D* test result was significantly negative), and *P. vivax*, except for SAM, MCA (only Tajima’s *D* test results were significantly negative for both) and EAS (Table 2). Likewise, separate country-specific analyses yielded significantly negative Tajima’s and Fu’s test values for *P. falciparum* populations from Brazil and Venezuela (Supplementary Table 9) and *P. vivax* populations from Brazil, Colombia, and Peru (Supplementary Table 10). In addition, the frequency distributions of pairwise nucleotide mismatches in mitochondrial sequences fit those expected under a sudden demographic expansion model for most regional populations of *P. falciparum*, except for AFR and SOA (Supplementary Fig. 4 and Supplementary Table 11), and all populations of *P. vivax* (Supplementary Fig. 5 and Supplementary Table 11). Evidence for demographic expansion was further obtained in country-specific mismatch distribution analyses of SAM populations of *P. falciparum* (Supplementary Fig. 6 and Supplementary Table 12) and *P. vivax* (Supplementary Fig. 7 and Supplementary Table 12).

**Table 2:** Results of Tajima’s *D* and Fu’s *F*_s_ neutrality tests applied to global and regional populations of *Plasmodium falciparum* and *P. vivax*. Statistically significant *P* values are underlined.

Population	*P. falciparum*				*P. vivax*			
	Tajima’s *D*	*P value*	Fu’s *F*_*s*_	*P value*	*Tajima’s D*	*P value*	Fu’s *F*_*s*_	*P value*
AFR^a^	−2.78152	<0.0001	−14.28997	<0.02	−2.73599	<0.0001	−6.32855	<0.02
CAM^b^	1.29320	0.889	−1.31251	<0.10	−2.14553	<0.0001	−4.08699	<0.02
EAS^c^	—	—	—	—	−0.98766	0.15	−1.74506	>0.10
MCA^d^	—	—	—	—	−1.90704	0.01	−3.05478	<0.05
MEL^e^	−2.10716	0.003	−4.63388	<0.02	−2.32606	<0.0001	−4.11114	<0.02
SAM^f^	−2.61638	<0.0001	−8.87358	<0.02	−2.53550	<0.0001	0.03668	>0.10
SEA^g^	−2.21823	<0.0001	−6.27935	<0.02	−2.16371	<0.0001	−6.61222	<0.02
SOA^h^	−1.89148	0.008	−2.88087	<0.05	−2.55500	<0.0001	−5.32889	<0.02
**Total**	**−2.68819**	**<0.0001**	**−18.2203**	**<0.02**	**−2.61983**	**0.001**	**−7.09593**	**<0.02**

^a^AFR = Africa; ^b^CAM = Central America (including Mexico in the *P. vivax* analyses); ^c^EAS = East Asia (only *P. vivax*); ^d^MCA = Middle East and Central Asia (only *P. vivax*); ^e^MEL = Melanesia; ^f^SAM = South America; ^g^SEA = Southeast Asia; and ^h^SOA = South Asia.

Moreover, Bayesian skyline analysis revealed evidence of a past *P. falciparum* population expansion worldwide between 25,000 and 10,000 years ago (Supplementary Fig. 8). Likewise, the global *P. vivax* population has expanded exponentially between 30,000 and 10,000 years before present (Supplementary Fig. 9). Evidence for past demographic expansions has also been found for the SAM population of both *P. falciparum* and *P. vivax*, but not for the CAM population of *P. vivax* (Supplementary Figs 10 and 11). Interestingly, neither *P. falciparum* nor *P. vivax* SAM populations show evidence of a decline in effective population size following their relatively recent arrival in the New World. In fact, present-day malaria parasites from this continent display clear signatures of expansion approximately 10,000–25,000 years ago; this expansion was experienced by the ancestral populations from which they have derived more recently (Supplementary Figs 10 and 11).

The TMRCA estimates for SAM populations of *P. falciparum* (37,002 years; 95% HPD interval, 21,385–56,606 years before present) and *P. vivax* (52,149 years; 95% HPD interval, 29,896–60,659 years before present) indicate that the hypothetical most recent common ancestor of New World malaria parasites largely predates the first human migrations to the continent. Moreover, these estimates are rather similar to those obtained for populations of AFR *P. falciparum* (35,384 years; 95% HPD interval, 21,101–54,974 years before present) and *P. vivax* (41,685 years; 95% HPD interval, 28,630-57,563 years before present). Therefore, skyline analysis and TMRCA estimates argue against a severe population bottleneck associated with the recent malaria parasite migration to the Americas (see also^14^); to the contrary, SAM lineages appear to have retained much of the diversity that preexisted in their ancestral populations.

### Parasite migration models

**Figure 4:**
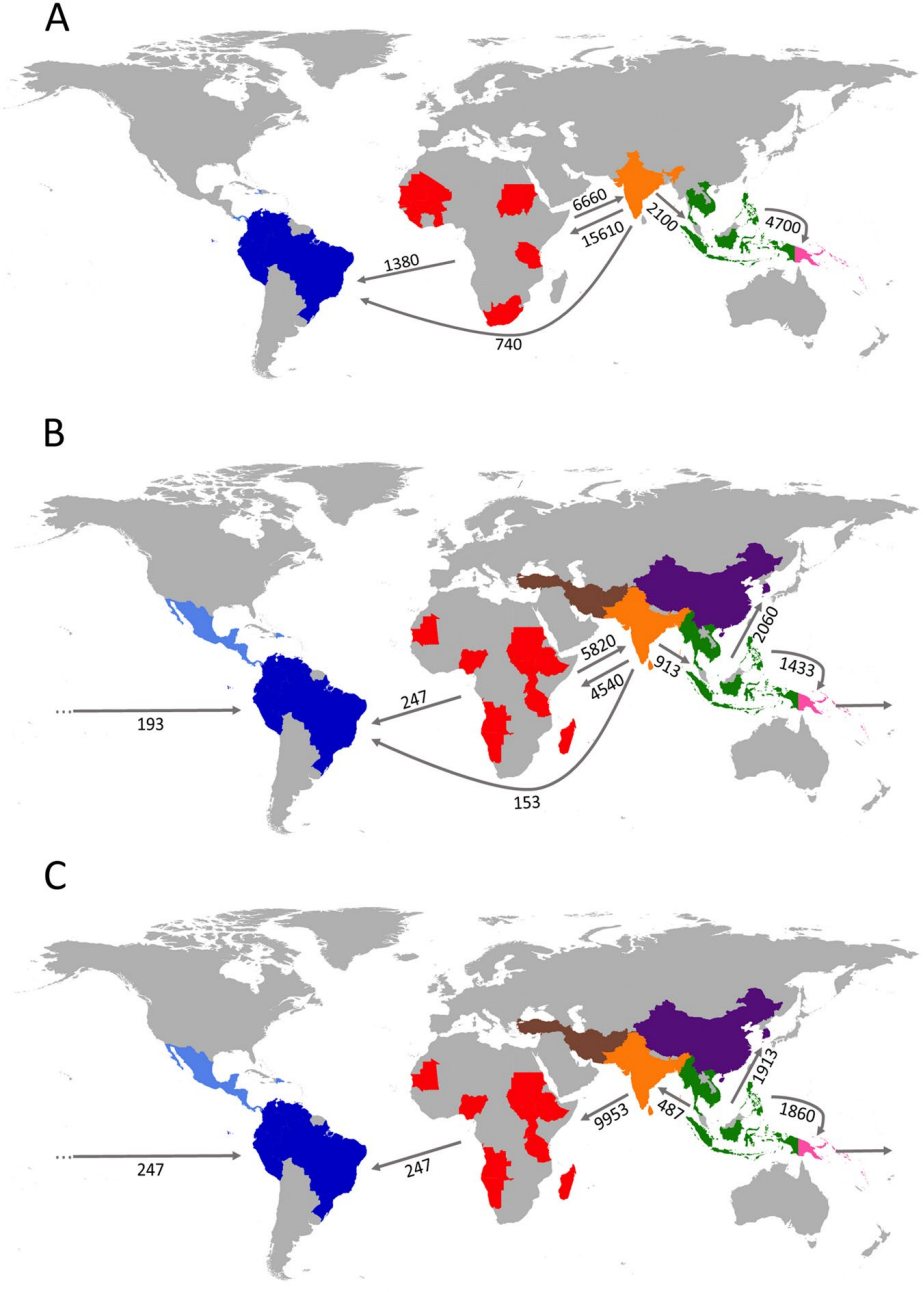
Magnitude and directionality of historical gene flow between regional populations of *Plasmodium falciparum* (**A**) and *P. vivax* (**B** and **C**). Estimates of median mutation-scaled pairwise migration rates obtained with the best-supported migration model for each species are shown next to the arrows. Migration models tested are described in Supplementary Fig. 13 and compared in Supplementary Tables 14 and 15; the major difference between the models shown in B and C is that the former assumes an out-of-Africa spread of *P. vivax*, whereas the latter assumes a Southeast Asian origin of this parasite. The geographic origins of mitochondrial lineages are indicated on the map at the country level using the same color code as those of Fig. 1 (for *P. falciparum*) and Fig. 2 (for *P. vivax*) to represent geographic regions. Maps were built using the open-access R software library *rworldmap: mapping global data* combined with the *ggplot2* library, which are both available at http://www.R-project.org/ (R Core Team, R: A Language and Environment for Statistical Computing.Vienna, Austria: R Foundation for Statistical Computing, 2017).

We used a Bayesian approach^29^ to estimate median mutation-scaled pairwise migration rates (*M*) and compare a priori parasite migration models. These models treat SAM and CAM as a single American population (“SAM and CAM combined”). The basic models assume an African origin of malaria parasites and subsequent eastward spread to Asia and MEL with separate westward migration from AFR to the Americas. However, models differ according to the potential genetic contribution of regional parasite populations (other than the African population) to New World populations of *P. falciparum* and *P. vivax*. The best-supported *P. falciparum* model (C1, which has a posterior probability of 0.75; Supplementary Fig. 12 and Supplementary Table 13) assumes gene flow mostly from AFR (median *M* = 1380.0) but also from SOA (median *M* = 740.0) to the Americas with intense bilateral gene flow between the AFR and SOA populations; no further genetic contribution to American lineages was observed from other regional populations (Fig. 4A). Model A1, which assumes gene flow to the New World from AFR, but not from SOA, is moderately supported (posterior probability = 0.24).

The *P. vivax* a priori migration models treated MCA and SOA as a single population (SOA*) and included *Plasmodium vivax*/*P. simium* samples from the Atlantic Forest in the American population (SAM and CAM combined). The best-supported *P. vivax* model assuming an African origin of *P. vivax* (F, which has a posterior probability of 0.92; Supplementary Fig. 13 and Supplementary Table 14), similar to the top *P. falciparum* model, allows unilateral gene flow to the New World from both AFR (median *M* = 246.7) and SOA* (median *M* = 153.3) and assumes bilateral gene flow between AFR and SOA*. However, in contrast to the *P. falciparum* migration models, this *P. vivax* model also assumes a unidirectional gene flow from MEL to the Americas (median *M* = 193.3; Fig. 4B). The next best supported model assuming an African origin of *P. vivax* (A1, with a posterior probability of 0.05) allows for gene flow to the New World only from AFR, not from SOA* (Supplementary Fig. 13 and Supplementary Table 14).

We also modeled an alternative scenario that assumes a Southeast Asian (instead of African) origin of *P. vivax* with subsequent eastward spread to EAS and MEL and westward migration to South Asia, AFR, and the Americas (Supplementary Fig. 14). The best-supported model assuming a Southeast Asian origin of *P. vivax* (B, which has a posterior probability of 0.99; Supplementary Table 15) also allows for gene flow to the New World from both AFR and MEL (median *M* = 246.7 for both; Fig. 4C).

## Discussion

### Mitogenomes to track the migration of malaria parasites

There are clear advantages to using the 6-kb mitogenome to track past migrations of malaria parasites. First, because the mitogenome is uniparentally inherited through the female gamete, lineages do not recombine with each other and their genealogy can be resolved by phylogenetic analysis^30^. Moreover, mitochondrial SNPs appear to be evolutionarily neutral and enable an accurate description of the parasite’s demographic history^14,31^. Finally, since this genome can be easily sequenced with Sanger-based or next-generation sequencing methods, several hundreds of complete mitogenomes of *P. vivax* and *P. falciparum* collected worldwide are currently available in public databases. Some limitations, however, must be considered. First, mitogenomes provide little resolution for fine-scale studies of geographic polymorphism because of their relatively limited number of segregating sites. Second, because there is no recombination, mitochondrial sequence analysis provides a picture of a parasite′s single locus, which may not be representative of its entire genome. Third, our data violate some assumptions of the underlying coalescent model of migration analysis, such as high inbreeding and constant *N*_e_ and gene flow over time. Fourth, the assumed geographic origin of malaria parasites affects all other gene flow rates estimated by the models. Moreover, the undetermined genetic contribution from unsampled parasite populations (*e.g*., from Europe and other parts of AFR) may confound estimates of gene flow between sampled populations. Although simulation analyses suggest that coalescent-based migration models are very robust to biases introduced by small to moderate violations of model assumptions that are usually seen in real-world populations^32^, we consider these model outputs to be a first glimpse at the complex demographic history of human malaria parasites.

### Origins of *Plasmodium falciparum* circulating in the New World

*Plasmodium falciparum* is currently hypothesized to have originated from a lateral transfer from gorillas to humans in Western Africa between 10,000 and 100,000 years ago^33^. From the African cradle, the parasite spread to Eurasia and the southwest Pacific as modern humans colonized these regions^24^, whereas a separate, massive human migration out of AFR brought *P. falciparum* to the New World^8^.

More than 7 million enslaved Africans arrived in the Americas over three centuries. More than 2 million were disembarked at Spanish ports in the West Indies, Veracruz (Mexico), and Cartagena (Colombia), whereas nearly 5 million were brought to the main Portuguese-run ports of Brazil, Recife, Salvador, and Rio de Janeiro^34^. Most slaves imported to Brazil throughout the 16^th^ century came from the Upper Guinea Coast (present-day Senegal and Gambia). Angola and Congo became the major source of slaves in the late 16^th^ century, complemented by the Mina Coast (Togo, Benin, and southwestern Nigeria) starting in the early 1700s and by southeastern Africa (Mozambique and Madagascar) from the end of the 18^th^ century. Nearly 2 million Africans are estimated to have been brought to Brazil during the 18^th^ century^35^. Enslaved Africans were captured not only along the coast, where major European outposts were located, but also in more distant Central or East African locations^35^.

Therefore, *P. falciparum* lineages introduced by slave trade into the Americas originated from a wide variety of African locations and resulted in additional genetic contribution, as suggested by the best-supported migration model of SOA migrants crossing the Indian Ocean to coastal areas and islands (*e.g*., Madagascar) of Southeast Africa (Fig. 4A). The lack of an apparent bottleneck in South American populations of *P. falciparum* is somewhat surprising. Not only parasite migration events, but also selective sweeps induced by large-scale antimalarial use in more recent years could have drastically reduced parasite diversity in this continent. We argue, however, that the current diversity levels have originated from the very many introductions of this parasite into the continent over centuries, from several different source populations in Africa. The substantial differentiation between present-day SAM and AFR lineages of *P. falciparum* likely results from the fact that we have not sampled all potential AFR source populations (Supplementary Fig. 15) or all *P. falciparum* subpopulations currently found in the Americas. Moreover, since not all migrants are expected to have successfully adapted to local vectors, the parasite populations currently found in the Americas are not expected to represent a random subsample of their multiple source populations. The relative contribution of European lineages to SAM and CAM populations of *P. falciparum* cannot be estimated from our data because this potential source population remains largely unsampled.

### Early human migration and the origins of *P. vivax* diversity in the New World

The extensive genetic diversity currently found in *P. vivax* populations in the Americas is consistent with successive migratory waves and subsequent admixture between parasites from different source populations^14^. Our data suggest that the extant lineages of AFR and SOA* populations represent major genetic contributors to current New World lineages of *P. vivax* (Fig. 4B); again, the relative contribution of European lineages remains undetermined.

Carter^3^ has speculated that a relapsing parasite such as *P. vivax* might have survived long-range, pre-Columbian oceanic crossings from the Western Pacific to the Americas through a reverse Kon-Tiki route. Accordingly, the best-supported *P. vivax* migration models assume a significant gene flow from MEL to the Americas (Fig. 4B,C) in addition to the expected genetic contribution from AFR and SOA* populations. Indeed, recent genome-wide analyses of human populations have revealed that at least three South American Native peoples – the Suruí, Karitiana, and Xavante – share more ancestry with indigenous populations from Australia, Melanesia, and island SEA than with Eurasians or other Native Americans^36^. These findings are consistent with two founding populations – one from Eurasia and one from Australasia – entering the Americas. This second founding population consisted of descendants of “population Y”, which is hypothesized to have contributed genes to both Australasians and Native Americans. Traces of Australasian ancestry were also independently found in the nuclear genomes of Aleutian islanders and Athabascans from North America and Suruí from Brazil^37^ and in the mitogenome of now-extinct Botocudo peoples from Brazil^38^.

One can hypothesize that descendants of population Y might have carried Melanesian strains of *P. vivax* to SAM (and possibly to the Amazon) long before the Europeans arrived, but could malaria parasites have survived in small, impermanent, and relatively isolated villages found in the Amazon in pre-Columbian times? This notion of a sparsely populated, pristine Amazon^39^ is contradicted by emerging evidence for the existence of large sedentary societies across this region that predated European conquest^40–42^. Evidence of these relatively complex societies has been found only 300–500 km away from the reserves where the Suruí and Karitiana peoples, who bear clear genetic traces of Australasian ancestry, currently live in the Western Amazon of Brazil^43,44^.

### **The***P. simium***puzzle**

The evolutionary history of *P. vivax* is complex. This human lineage appears to have originated as part of the radiation of non-human primate malaria parasites that are now commonly found in SEA^45^. However, closely related parasites have been found to infect chimpanzees and gorillas in sub-Saharan Africa^28,46^, which is consistent with an early introduction of ancient parasite lineages into AFR that infected hominids and may have given rise to the human parasite species known as *P. vivax*. Surprisingly, a *P. vivax*-like parasite can also be found in New World platyrrhine monkeys, which diverged from African great apes and Old-World catarrhine monkeys more than 40 million years ago^47^. *Plasmodium simium* was originally described in non-human primates from São Paulo, southeast Brazil^48^, and subsequently found in two genera of the Atelidae family, howler monkeys (*Alouatta caraya* and *A. clamitans*) and woolly spider monkeys (*Brachyteles arachnoides*)^49^. More recently, natural *P. simium* infections have also been described in capuchin monkeys (*Cebus* and *Sapajus* species of the Cebinae subfamily of the Cebidae family)^50^ and the black-fronted titi monkey *Callicebus nigrifrons* (Callicebinae subfamily of the Pitheciidae family)^51^ from the Atlantic Forest of Brazil.

*Plasmodium simium* is morphologically and genetically indistinguishable from *P. vivax*^49,52,53^, which is consistent with one or more host switches between humans and monkeys in recent evolutionary times. The direction of the host switch (whether from monkeys to humans or vice-versa) remains under debate^47,54^, although available phylogenetic data renders a lateral transfer from New World monkeys to humans very unlikely^31,55,56^. By comparing levels of genetic diversity in *P. vivax* and *P. simium* populations from the Atlantic Forest, one can infer that the species with the greatest polymorphism is likely to be the ancestor^47,54^. Our findings strongly support a recent human-to-monkey transfer in Brazil, which is consistent with the low diversity of *P. simium* lineages recovered from monkeys living in three separate locations of this country (only two haplotypes found) compared with *P. vivax* strains from humans living in the Atlantic Forest ecosystem and in the Amazon.

Moreover, a comparison of newly available *P. simium* samples with worldwide *P. vivax* isolates allowed us to test further hypotheses about the geographic origin of the *P. vivax*-like parasites that currently infect monkeys in Brazil. Li and colleagues^12^ characterized genetic polymorphisms in the S-type of the *18S rRNA* gene and the plastid genome that putatively distinguish Old World from New World lineages of *P. vivax*. Furthermore, they found that the Fonseca MRA-353 strain of *P. simium* carried the Old World-type sequence and speculated that *P. simium* and the *P. vivax* populations that currently circulate among humans in the Americas entered the continent on two separate occasions that were most likely from different source populations^12^. Cormier^13^ further hypothesized that East Asian migrants might have introduced Old World *P. vivax*/*P. simium* lineages into the Atlantic Coast of Brazil in the early 1800s. However, subsequent analyses revealed both New World and Old-World types of *18 S rRNA* gene sequences in parasites from the Amazon Basin of Brazil and from Sri Lanka (Supplementary Text 1). Moreover, the *P. simium* lineages *Atl1* and *Atl2* do not cluster with Old World *P. vivax* populations in our phylogenetic analyses (Fig. 2A,B). We thus conclude that both Old World- and New World-type *18 S rRNA* gene sequences are found in SAM and SOA and that *P. simium* mitogenomes are not closely related to Old World mitochondrial lineages of *P. vivax*.

Significantly, ATL parasites (including *Atl1* and *Atl2*) are transmitted by anopheline mosquitoes of the *Kerteszia* subgenus, mainly *Anopheles K. cruzi* and *An. K. bellator*, which breed in water trapped by the leaf axils of bromeliad plants in the Atlantic Forest^57^. Conversely, *P. vivax* populations circulating among humans in the Amazon Basin are transmitted by members of *Nyssorhynchus* subgenus, mainly *An. darlingi*^58^. The adaptation to different local mosquito vectors may have favored the divergence between *P. vivax* lineages that have been brought to Brazil and currently circulate in distinct, non-contiguous endemic foci in the Amazon and along the Atlantic Coast. Accordingly, an example of genetically distinct *P. vivax* subpopulations being transmitted by different vectors has been described in southern Mexico^59^. We hypothesize that a few ATL lineages adapted to *Kerteszia* anophelines have been transferred to platyrrhine monkeys, favored by their vectors’ ability to bite both at the canopy of the trees (where monkeys live) and at the ground level (where humans are commonly found)^49^. The relative role of these zoonotic lineages as a source of human infections in coastal Brazil is a matter that requires further investigation in the context of ongoing malaria elimination efforts in the region^28^.

## Materials and Methods

### Ethics statement

Study protocols were approved by the Institutional Review Board of the Institute of Biomedical Sciences, University of São Paulo (protocol #1102), by the Institutional Review Board of the Papua New Guinea Institute of Medical Research (IRB 0919), and by the Government of Papua New Guinea Medical Research Advisory Committee (MRAC 09/24). Written informed consent was obtained from all adult patients, and minors (<18 years of age) had consent provided by a parent/guardian on their behalf. Monkey-derived blood samples were collected under the approval of the Brazilian Institute of Environment and Renewable Natural Resources (protocol #54173-1). All the methods were carried out in accordance with the approved guidelines.

### Parasite samples and DNA sequencing

We generated new full-length mitogenome sequences from 380 parasite isolates collected between 2001 and 2013, comprising 244 (64.2%) *P. falciparum* isolates (147 from Brazil, 21 from Venezuela, 6 from Panama, 27 from Tanzania, 20 from Indonesia, and 23 from Papua New Guinea), 127 (33.4%) *P. vivax* isolates (77 from Brazil, 32 from Panama, and 52 from Papua New Guinea), and 9 (2.4%) isolates of the *P. vivax*-like monkey parasite *P. simium* from the Atlantic Forest of South and Southeast Brazil.

DNA templates were prepared from 200 µL of whole blood samples using QIAamp DNA Blood Mini Kits (Qiagen, Hilden, Germany). We designed primer pairs to amplify overlapping fragments of the mitogenomes of *P. falciparum*, *P. vivax*, and *P. simium*; primer sequences are given in Supplementary Table 16. Long-range, high-fidelity PCR amplification (amplicon size range, 1147–3497 bp) was performed using PrimeSTAR DNA polymerase (Takara, Otsu, Shiga, Japan), which has efficient 3′ → 5′ exonuclease proof-reading activity. PCR was performed for all species in 100-μl total reaction volume containing 6 μl of DNA template, 0.3 μM concentrations of each oligonucleotide primer, 10× PrimeSTAR PCR Buffer, 2.5 mM concentrations of each deoxynucleoside (dNTP), and 2.5 units of PrimeSTAR DNA polymerase. PCR was performed on a GeneAmp PCR 9700 thermal cycler (Applied Biosystems, Foster City, CA) at 94 °C for 1 min, followed by 30 cycles of 98 °C for 10 sec, 55 °C for 5 sec, and 72 °C for 2 min. A final extension was done at 72 °C for 10 min. PCR products were purified with an Illustra GFX PCR and Gel Band Purification kit (GE Healthcare Biosciences, Pittsburgh, PA) and sequenced using the BigDye kit version 3.1 on an ABI 3130 DNA sequencer (Applied Biosystems, Foster City, CA). We used 13 internal oligonucleotide primer pairs to sequence both strands of each *P. falciparum* and *P. vivax* amplicon with reads ranging between 450 bp and 530 bp (*P. falciparum*) and 303 bp and 566 bp (*P. vivax*). The *P. simium* mitogenomes were amplified and sequenced with the *P. vivax* primer set and an additional set of five external and 10 internal primer pairs that were designed to match the only publicly available *P. simium* mitogenome sequence, which was derived from the Fonseca MRA-353 isolate^55^ (accession number, AY722798). All fragments amplified with either primer set were sequenced. Nucleotide ambiguities were removed by further confirming some sequences on two independent PCR reactions on the same DNA template. The sequenced fragments were filtered for quality and assembled into complete mitochondrial sequences (length, 5,884 bp for *P. falciparum* and 5,990 bp for *P. vivax*/*P. simium*) using the phred/phrap/consed software (http://www.phrap.org/phredphrapconsed.htm) and deposited in the GenBank database under accession numbers KY923289-KY923647.

### *Plasmodium falciparum* mitogenomes assembled from short sequence reads

We used publicly available whole-genome paired-end Illumina reads generated from *P. falciparum* isolates from AFR^60,61^ and SEA^60^ with known geographic origin were known (total of 925 short-read data sets) that were retrieved from the European Nucleotide Archive (ENA) of the European Molecular Biology Laboratory (EMBL) (accession numbers, ERS010434-ERS010659 and ERS041968-ERS041991). Sequence reads (average size, 100 bp) that mapped onto the mitochondrial DNA of the 3D7 reference strain (accession number, AY282930) were identified using BLASTN (https://blast.ncbi.nlm.nih.gov/Blast.cgi) and filtered using the fastq_quality_filter software of the FastX-Toolkit package (http://hannonlab.cshl.edu/fastx_toolkit/index.html); only reads with quality scores ≥15 in every nucleotide were further considered. Mitogenomes were assembled using GS Assembler software (Newbler) version 2.7 with a minimum level of coverage of 10 × (average coverage of 200×) for each nucleotide. These sequence reads have a potential issue with mixed infections, as they come from clinical isolates that may comprise multiple genetically distinct clones. There is therefore a risk of misassembling several different mitochondrial sequences into a single hybrid mitogenome. To prevent this, we first converted heterozygote calls to the majority allele if ≥75% of the reads in that sample were the majority allele (i.e., when the dominant clone could be clearly defined); otherwise, the sample was excluded from further analysis. Next, all *P. falciparum* mitogenomes retrieved from short sequence reads were analyzed for missassembling that could simulate historical recombination events. For this purpose, we applied five non-parametric recombination detection methods (MAXCHI, CHIMAERA, GENECONV, BOOTSCAN, and SISCAN) with their default parameters; they were implemented in the RPD3 computer program^62^. None of the methods revealed statistical evidence of recombination in any assembled mitogenome. Of the 925 short-read data sets retrieved from ENA, 812 (87.8%) generated mitogenome assemblings that were used in further analyses.

### Sources of additional mitogenomes

We additionally analyzed 739 complete or nearly complete *P. falciparum* mitogenomes deposited in the GenBank database from isolates from AFR, Americas, South Asia, SEA, and MEL (accession numbers, AY283018–AY282924; AB570434–AB570542; AB570544–AB570765, AB570767–AB570951; KJ569502–KJ569459; AJ276845–AJ276847; and KT119847–KT119883), 822 *P. vivax* complete or nearly complete mitogenomes from Africa, Americas, MCA, South Asia, SEA, EAS, and MEL (accession numbers, AY791517–AY791692, AY598035–AY598140, AB550270–AB550280, JN788737–JN788776, DQ396547–DQ396548, KC330370–KC330678, JQ240429–JQ240331, KF668442–KF668430, and KF668429–KF668361), and a single complete *P. simium* mitogenome from Brazil (accession number, AY722798). The final data set comprised 1,795 mitogenomes of *P. falciparum*, 931 of *P. vivax*, and 10 of *P. simium* that were aligned using MEGA version 6.0^63^ and edited by hand. Gap stripping left 5,775 sites analyzable in the *P. falciparum* alignment and 5,812 sites in the *P. vivax*/*P. simium* alignment. Supplementary Database 1 gives a list of all sequences analyzed in this study, along with their countries of origin and GenBank accession numbers, and Supplementary Fig. 2 shows the geographic locations of the SAM and CAM sample collection sites.

### Within-population diversity and between-population divergence

We used DnaSP software version 5^64^ to calculate the haplotype diversity (*H*), the standardized number of segregating sites, θ_*S*_^65^, and the average number of pairwise nucleotide differences per site, π^66^, which was calculated with the Jukes-Cantor’s correction. Arlequin software version 3.5^67^ was used to carry out Tajima’s^68^ and Fu’s *F*_s_^69^ neutrality tests, which detect deviations from a neutral evolution model that assumes random mating, no recombination, mutation-drift equilibrium, infinite sites, and constant population size. The statistical significance of both tests was examined using 1,000 coalescent simulations under a standard model of neutral evolution. A *P* value of 0.02 for Fu’s *F*_s_ test obtained from coalescent simulations corresponds to the conventional *P* value of 0.05^69^; therefore, Fu’s *F*_s_ test results were considered significant if they were associated with a *P* value < 0.02.

We also used Arlequin 3.5 software to estimate the Wright’s fixation index *F*_ST_, a measure of divergence between populations due to genetic structure^70^. Significance was evaluated using one-sided permutation tests with 1,000 simulations. The following regional populations were considered in *F*_ST_ calculations: AFR, SAM, CAM (including Mexico in *P. vivax* analyses; CAM), MCA; only for *P. vivax*, SOA, SEA, EAS (only for *P. vivax*), and MEL.

### Phylogeny and historical demography

Bayesian phylogenetic analysis was carried out separately for *P. falciparum* and *P. vivax*/*P. simium* using MrBayes version 3.2.1^71^ with two runs of four chains each, three heated and one cold, for seven million (*P. vivax*) or 10 million generations (*P. falciparum*). The trees were drawn using Dendroscope version 3.4 software^72^, and a color code was used to identify the geographic origin of parasites: AFR (red), SAM (dark blue), CAM (light blue), Atlantic Forest from southeast Brazil (ATL [yellow]; only for *P. vivax*/*P. simium*); MCA ([brown]; only for *P. vivax*), SOA (orange), SEA (green), EAS ([dark purple]; only for *P. vivax*), and MEL (pink).

Median-joining phylogenies^73^ were generated using Network version 4.6 (Fluxus Technologies, http://www.fluxu-engeneering.com) with the default parameters and transversions weighted twice as much as transitions. This analysis aimed to reconstruct global haplotype networks of the entire sets of *P. falciparum* and *P. vivax*/*P. simium* mitogenomes, and the same color code described above was used to show the geographic origins of samples. Straight lines connect pairs of haplotypes that differ by a single mutational step. Separate haplotype networks were also generated for parasites from the Americas to further explore regional patterns of genetic variation.

We used Arlequin 3.5 to obtain frequency distributions of pairwise mismatches between mitochondrial sequences to explore the demographic history of parasite populations. Multimodal, ragged distributions are expected under constant population size, whereas unimodal distributions are typically observed in expanding populations. Separate analyses were carried out for parasites from AFR, SAM (comprising ATL), CAM, MCA (only for *P. vivax*), SOA, SEA, EAS (only for *P. vivax*), and MEL. We also separately analyzed country-level parasite populations from the Americas (Brazil and Venezuela for *P. falciparum*; Brazil, Colombia, and Peru for *P. vivax*). We calculated the sum of square deviations (SDD) and the raggedness index (R) to compare observed mismatch distributions with those expected under a sudden demographic expansion model^74^. These analyses were carried out using the *pegas* package of R software version 3.3.0 with 1,000 pseudoreplicates.

Next, we used the Markov chain Monte Carlo (MCMC) method implemented in BEAST software version 1.7.2^75^ to fit Bayesian skyline coalescent models that allowed us to track changes in *N*_e_ over time and estimate the time TMRCA of mitochondrial lineages. Separate analyses were run for the global population of each species and for the following regional populations: AFR, SAM (comprising ATL), CAM (only for *P. vivax*), MCA (only for *P. vivax*), SOA, SEA, EAS (only for *P. vivax*), and MEL. A Hasegawa-Kishino-Yano (HKY) nucleotide substitution model was used. Analyses were run for 200 million generations; the first 20,000 generations were discarded as burn-in, and sampling was performed every 5,000 generations. We used a strict molecular clock with 12 × 10^−9^ substitutions per site per year as the average nucleotide substitution rate for mitogenomes of malaria parasites, derived from a comparison of the rate for cytochrome b evolution in avian malaria parasites relative to its rate in bird hosts, for which dated fossils are available^76^. Note that this nucleotide substitution rate is substantially (1.5 to 4 times) faster than previous estimates based on assumed host-parasite co-speciation events^14,77^. In addition, a quite recently published analysis estimates mitochondrial nucleotide substitution rates at 3.8 × 10^−9^ substitutions per site per year for mammalian malaria parasites^78^. We therefore note that parasite lineages currently found in the Americas may be up to four times older than estimated here, depending on the molecular clock used. BEAST outputs were visualized with Tracer version 1.5 software^79^. We used TreeAnnotator version 2.1.2^79^ to obtain consensus trees derived from the skyline analyses after discarding the first 20,000 trees as burn-in. TMRCA estimates (with 95% HPD intervals) were obtained for the branches with a posterior probability >0.70 in the consensus trees. The topology of these consensus trees was remarkably similar to that of the Bayesian phylogenetic trees generated by MrBayes.

### Parasite migration models

MIGRATE-N version 3.6.11 software^29^ was used to compare parasite migration models and make inferences regarding the sources of malaria parasites currently found in the Americas. MIGRATE-N uses MCMC simulations to sample possible coalescent genealogies and to estimate, using a Bayesian approach, two sets of parameters: (a) mutation-scaled effective population sizes (θ = 4*Ν*_e_μ, where *N*_e_ is the effective population size and μ is the mutation rate) and (b) mutation-scaled pairwise migration rates (*M* = *m*/μ, where *m* is the migration rate). MIGRATE-N also provides the marginal likelihood of each migration model. The underlying coalescent model assumes neutral evolution, constant migration rates and population sizes over *N*_e_ generations (in haploid organisms) and that all potential source populations have been sampled^80^. Log marginal likelihoods (log mL) calculated by thermodynamic integration with Bézier approximation^81^ were used to rank models.

We compared 8 a priori models for *P. falciparum* that differ in the presence and directionality of gene flow between particular pairs of populations (Supplementary Fig. 12). Model A assumes an African origin of *P. falciparum*^33^ and its subsequent unidirectional, eastward spread out of Africa, first to South Asia and then to SEA and MEL^24,82^. This parasite is assumed to have been introduced separately into the Americas (SAM and CAM combined) from the AFR population^2,8^. The next model (A1) is identical to A, but it assumes a bidirectional flow between AFR and SOA populations. The next six models (B, B1, C, C1, D, and D1) assume additional contributions of either SOA, SEA, or MEL to the extant diversity of New World *P. falciparum* populations.

Likewise, we compared 11 different *P. vivax* models assuming an African origin of *P. vivax*^46^. As with *P. falciparum*, model A assumes a stepwise out-of-AFR spread to the Old World and a separate migration of the AFR population to the Americas (SAM and CAM combined; Supplementary Fig. 13). Model A1 assumes a bidirectional gene flow between AFR and the MCA and SOA populations (the latter two merged into a single population, SOA*) under the hypothesis that some major *P. vivax* lineages currently found in AFR have been reintroduced to this continent from these sources^3,26^. The next eight models (B, B1, C, C1, D, D1, E, and E1) assume additional gene flow to the New World from either SOA*, SEA, EAS, or MEL^3,15,16,28,58^. Finally, model F allows for a unilateral gene flow from SOA* and MEL populations to the Americas with bilateral gene flow between AFR and SOA*.

Next, we compared six models under an alternative scenario that assumes a Southeast Asian (instead of African) origin of *P. vivax*^28,31^ (Supplementary Fig. 14). The basic model A assumes a unidirectional eastward gene flow to EAS and MEL and a stepwise gene flow westward to South Asia, Africa, and the Americas. The next four models (B, C, D, and E) assume additional gene flow to the New World from either MEL, SOA*, SEA, or EAS, whereas model F assumes additional gene flow from both SOA* and MEL populations to the Americas.

For each *P. falciparum* migration model, we ran two replicates of four parallel static chains with temperatures of 1.0, 1.5, 3.0 and 10^6^ and a swapping interval of 1. We used uniform priors between 0 and 0.15 (delta = 0.013) for θ and between 0 and 30,000 (delta = 3,000) for *M*. In each run, we discarded 30,000 trees as burn-in and recorded 5 × 10^6^ steps with an increment of 10. *P. vivax* models were run with similar settings, except that *M* priors ranged between 0 and 20,000 (delta = 2,000). We assessed model convergence by examining the posterior distributions of the parameters to determine whether they were unimodal with smooth curves. Natural log Bayes factors (LBF) were calculated as a ratio of the marginal likelihoods to calculate probabilities of each model. LBF <−2 indicate a strong preference for the best-supported model.

### Data availability statement

All data used in the current analyses have been deposited into public databases.

## Electronic supplementary material

Supplementary Information

Supplementary Dataset 1

## References

[1] Harcourt AH (2016). Human phylogeography and diversity. Proc. Natl. Acad. Sci. USA.

[2] Bruce-Chwatt LJ (1965). Paleogenesis and paleo-epidemiology of primate malaria. Bull. World Health Organ..

[3] Carter R (2003). Speculations on the origins of *Plasmodium vivax* malaria. Trends Parasitol..

[4] Kwiatkowski DP (2005). How malaria has affected the human genome and what human genetics can teach us about malaria. Am. J. Hum. Genet..

[5] McNeill, W. H. *Plagues and Peoples* (Anchor Press/Doubleday, 1976).

[6] Joralemon D (1982). New world depopulation and the case of disease. J. Anthropol. Res..

[7] de Castro MC, Singer BH (2005). Was malaria present in the Amazon before the European conquest?Available evidence and future research agenda. J. Archaeol. Sci..

[8] Yalcindag E (2012). Multiple independent introductions of *Plasmodium falciparum* in South America. Proc. Natl. Acad. Sci. USA.

[9] Bruce-Chwatt, L. J. & de Zulueta, J. *The Rise and Fall of Malaria in Europe: A Historico-epidemiological Study* (Oxford Univ. Press, 1980).

[10] Cambournac FJ (1994). Contribution to the history of malaria epidemiology and control in Portugal and some other places. Parassitologia.

[11] Zimmerman PA, Ferreira MU, Howes RE, Mercereau-Puijalon O (2013). Red blood cell polymorphism and susceptibility to *Plasmodium vivax*. Adv. Parasitol..

[12] Li J (2001). Geographic subdivision of the range of the malaria parasite *Plasmodium vivax*. Emerg. Infect. Dis..

[13] Cormier LA (2010). The historical ecology of human and wild primate malarias in the New World. Diversity.

[14] Taylor JE (2013). The evolutionary history of *Plasmodium vivax* as inferred from mitochondrial genomes: parasite genetic diversity in the Americas. Mol. Biol. Evol..

[15] Winter DJ (2015). Whole genome sequencing of field isolates reveals extensive genetic diversity in *Plasmodium vivax* from Colombia. PLoS Negl. Trop. Dis..

[16] Hupalo DN (2016). Population genomics studies identify signatures of global dispersal and drug resistance in *Plasmodium vivax*. Nat. Genet..

[17] de Oliveira TC (2017). Genome-wide diversity and differentiation in New World populations of the human malaria parasite *Plasmodium vivax*. PLoS Negl. Trop. Dis..

[18] Gerszten E, Allison MJ, Maguire B (2012). Paleopathology in South American mummies: a review and new findings. Pathobiology.

[19] Bianucci R, Araújo A, Pusch CM, Nerlich AG (2015). The identification of malaria in paleopathology-An in-depth assessment of the strategies to detect malaria in ancient remains. Acta Trop..

[20] Boyd MF (1941). An historical sketch of the prevalence of malaria in North America. Am. J. Trop. Med. Hyg..

[21] Wood CS (1975). New evidence for a late introduction of malaria into the New World. Curr. Anthropol..

[22] Carter R, Mendis KN (2002). Evolutionary and historical aspects of the burden of Malaria. Clin. Microbiol. Rev..

[23] Joy DA (2003). Early origin and recent expansion of *Plasmodium falciparum*. Science.

[24] Tanabe K (2010). *Plasmodium falciparum* accompanied the human expansion out of Africa. Curr. Biol..

[25] Gelabert P (2016). Mitochondrial DNA from the eradicated European *Plasmodium vivax* and *P*. *falciparum* from 70-year-old slides from the Ebro Delta in Spain. Proc. Natl. Acad. Sci. USA.

[26] Culleton R, Carter R (2012). African *Plasmodium vivax*: distribution and origins. Int. J. Parasitol..

[27] Prugnolle F (2013). Diversity, host switching and evolution of *Plasmodium vivax* infecting African great apes. Proc. Natl. Acad. Sci. USA.

[28] Brasil P (2017). Outbreak of human malaria caused by Plasmodium simium in the Atlantic Forest in Rio de Janeiro: a molecular epidemiological investigation. Lancet Glob. Health.

[29] Beerli P (2006). Comparison of Bayesian and maximum-likelihood inference of population genetic parameters. Bioinformatics.

[30] Avise JC (1987). Intraspecific phylogeography: the mitochondrial DNA bridge between population genetics and systematics. Annu. Rev. Ecol. Syst..

[31] Cornejo OE, Escalante AA (2006). The origin and age of *Plasmodium vivax*. Trends Parasitol..

[32] Strasburg JL, Rieseberg LH (2010). How robust are “isolation with migration” analyses to violations of the im model? A simulation study. Mol. Biol. Evol..

[33] Liu W (2010). Origin of the human malaria parasite *Plasmodium falciparum* in gorillas. Nature.

[34] Emory University. *Voyages. The Trans-Atlantic Slave Trade Database* (Emory University, 2016).

[35] Rodrigues, J. H. *Brasil e África: Outro Horizonte* (Civilização Brasileira, 1982).

[36] Skoglund P (2015). Genetic evidence for two founding populations of the Americas. Nature.

[37] Raghavan M (2015). Genomic evidence for the Pleistocene and recent population history of Native Americans. Science.

[38] Gonçalves VF (2013). Identification of Polynesian mtDNA haplogroups in remains of Botocudo Amerindians from Brazil. Proc. Natl. Acad. Sci. USA.

[39] Meggers BJ (1954). Environmental limitation on the development of culture. Am. Anthropol..

[40] Heckenberger MJ (2008). Pre-Columbian urbanism, anthropogenic landscapes, and the future of the Amazon. Science.

[41] Heckenberger MJ (2009). Lost cities of the Amazon. Sci. Am..

[42] Carson JF (2014). Environmental impact of geometric earthwork construction in pre-Columbian Amazonia. Proc. Natl. Acad. Sci. USA.

[43] Pärssinen M, Schaan D, Ranzi A (2009). Pre-Columbian geometric earthworks in the upper Purús: a complex society in western Amazonia. Antiquity.

[44] Watling J (2017). Impact of pre-Columbian “geoglyph” builders on Amazonian forests. Proc. Natl. Acad. Sci. USA.

[45] Muehlenbein MP (2015). Accelerated diversification of nonhuman primate malarias in Southeast Asia: adaptive radiation or geographic speciation?. Mol. Biol. Evol..

[46] Liu W (2014). African origin of the malaria parasite *Plasmodium vivax*. Nat. Commun..

[47] Tazi L, Ayala FJ (2011). Unresolved direction of host transfer of *Plasmodium vivax* v. *P. simium* and *P. malariae v. P. brasilianum*. Infect. Genet. Evol..

[48] Da Fonseca F (1951). *Plasmodium* of a primate of Brazil. Mem. Inst. Oswaldo Cruz.

[49] Deane LM (1992). Simian malaria in Brazil. Mem. Inst. Oswaldo Cruz.

[50] de Alvarenga DAM (2015). Simian malaria in the Brazilian Atlantic forest: first description of natural infection of capuchin monkeys (Cebinae subfamily) by *Plasmodium simium*. Malar. J..

[51] Bueno, M. G. *Pesquisa de Leishmania spp. e Plasmodium spp. em Primatas Neotropicais Provenientes de Regiões de Mata Atlântica e Amazônia Impactadas por Ações Antrópicas: Investigação in situ e ex situ*. Doctoral dissertation (University of São Paulo, 2012).

[52] Leclerc MC (2004). Meager genetic variability of the human malaria agent *Plasmodium vivax*. Proc. Natl. Acad. Sci. USA.

[53] Lim CS, Tazi L, Ayala FJ (2005). *Plasmodium vivax*: recent world expansion and genetic identity to *Plasmodium simium*. Proc. Natl. Acad. Sci. USA.

[54] Rich SM (2004). The unpredictable past of *Plasmodium vivax* revealed in its genome. Proc. Natl. Acad. Sci. USA.

[55] Jongwutiwes S (2005). Mitochondrial genome sequences support ancient population expansion in *Plasmodium vivax*. Mol. Biol. Evol..

[56] Mu J (2005). Host switch leads to emergence of *Plasmodium vivax* malaria in humans. Mol. Biol. Evol..

[57] Marrelli MT, Malafronte RS, Sallum MA, Natal D (2007). *Kerteszia* subgenus of *Anopheles* associated with the Brazilian Atlantic rainforest:current knowledge and future challenges. Malar. J..

[58] Sinka ME (2010). The dominant *Anopheles* vectors of human malaria in the Americas: occurrence data, distribution maps and bionomic precis. Parasit Vectors.

[59] Joy DA (2008). Local adaptation and vector-mediated population structure in *Plasmodium vivax* malaria. Mol. Biol. Evol..

[60] Miotto O (2013). Multiple populations of artemisinin-resistant *Plasmodium falciparum* in Cambodia. Nat. Genet..

[61] Mobegi VA (2014). Genome-wide analysis of selection on the malaria parasite *Plasmodium falciparum* in West African populations of differing infection endemicity. Mol. Biol. Evol..

[62] Martin DP (2010). RDP3: a flexible and fast computer program for analyzing recombination. Bioinformatics.

[63] Tamura K, Stecher G, Peterson D, Filipski A, Kumar S (2013). MEGA6: molecular evolutionary genetics analysis version 6.0. Mol. Biol. Evol..

[64] Librado P, Rozas J (2009). DnaSPv5: a software for comprehensive analysis of DNA polymorphism data. Bioinformatics.

[65] Watterson GA (1975). On the number of segregating sites in genetical models without recombination. Theor. Popul. Biol..

[66] Nei, M. *Molecular Evolutionary Genetics* (Columbia University Press, 1987).

[67] Excoffier L, Lischer HE (2010). Arlequin suite ver 3.5: a new series of programs to perform population genetics analyses under Linux and Windows. Mol. Ecol. Resour..

[68] Tajima F (1983). Evolutionary relationship of DNA sequences in finite populations. Genetics.

[69] Fu YX (1997). Statistical tests of neutrality of mutations against population growth, hitchhiking and background selection. Genetics.

[70] Weir BS, Cockerham CC (1984). Estimating *F* statistics for the analysis of population structure. Evolution.

[71] Ronquist F (2012). MrBayes 3.2: efficient Bayesian phylogenetic inference and model choice across a large model space. Syst. Biol..

[72] Huson DH (2007). Dendroscope: an interactive viewer for large phylogenetic trees. BMC Bioinformatics.

[73] Bandelt HJ, Forster P, Rohl A (1999). Median-joining networks for inferring intraspecific phylogenies. Mol. Biol. Evol..

[74] Rogers AR, Harpending H (1992). Population growth makes waves in the distribution of pairwise genetic differences. Mol. Biol. Evol..

[75] Drummond AJ, Suchard MA, Xie D, Rambaut A (2012). Bayesian phylogenetics with BEAUti and the BEAST 1.7. Mol. Biol. Evol..

[76] Ricklefs RE, Outlaw DC (2010). A molecular clock for malaria parasites. Science.

[77] Bensch S (2013). How can we determine the molecular clock of malaria parasites?. Trends Parasitol..

[78] Andreína Pacheco M. *et al*. Mode and rate of evolution of haemosporidian mitochondrial genomes: timing the radiation of avian parasites. *Mol Biol Evol*. in press (2017). 10.1093/molbev/msx285 PMC5850713 29126122

[79] Drummond AJ, Rambaut A (2007). BEAST: Bayesian evolutionary analysis by sampling trees. BMC Evol. Biol..

[80] Kuhner MK (2009). Coalescent genealogy samplers: windows into population history. Trends Ecol. Evol..

[81] Beerli P, Palczewski M (2010). Unified framework to evaluate panmixia and migration direction among multiple sampling locations. Genetics.

[82] Tanabe K (2013). *Plasmodium falciparum* mitochondrial genetic diversity exhibits isolation-by-distance patterns supporting a sub-Saharan African origin. Mitochondrion.

