# Supplementary material for: Human migration and the spread of malaria parasites to the New World
Source: Sci Rep. 2018 Jan 31;8:1993. doi: 10.1038/s41598-018-19554-0 (PMC5792595; doi:10.1038/s41598-018-19554-0)
Supplement: Supplementary file 1 — Supplementary Information [file 41598_2018_19554_MOESM1_ESM.pdf]

Supplementary Figures, Tables, and Text

Human migration and the spread of malaria parasites to the New World

Priscila T. Rodrigues[1*], Hugo O. Valdivia[2†], Thais C. de Oliveira[1], João Marcelo P. Alves[1], Ana Maria R. C. Duarte[3], Crispim Cerutti-Junior[4], Julyana C. Buery[4], Cristiana F. A. Brito[5], Júlio César de Souza, Jr[6,7], Zelinda M. B. Hirano[6,7], Marina G. Bueno[8], José Luiz Catão-Dias[8], Rosely S. Malafronte[9], Simone Ladeia-Andrade[10], Toshihiro Mita[11], Ana Maria Santamaria[12], José E. Calzada[12], Indah S. Tantular,[13] Fumihiko Kawamoto[14], Leonie R. J. Raijmakers[15], Ivo Mueller[16,17], M. Andreina Pacheco[18], Ananias A. Escalante[18], Ingrid Felger[19,20*] and Marcelo U. Ferreira[1*]

Supplementary Figures

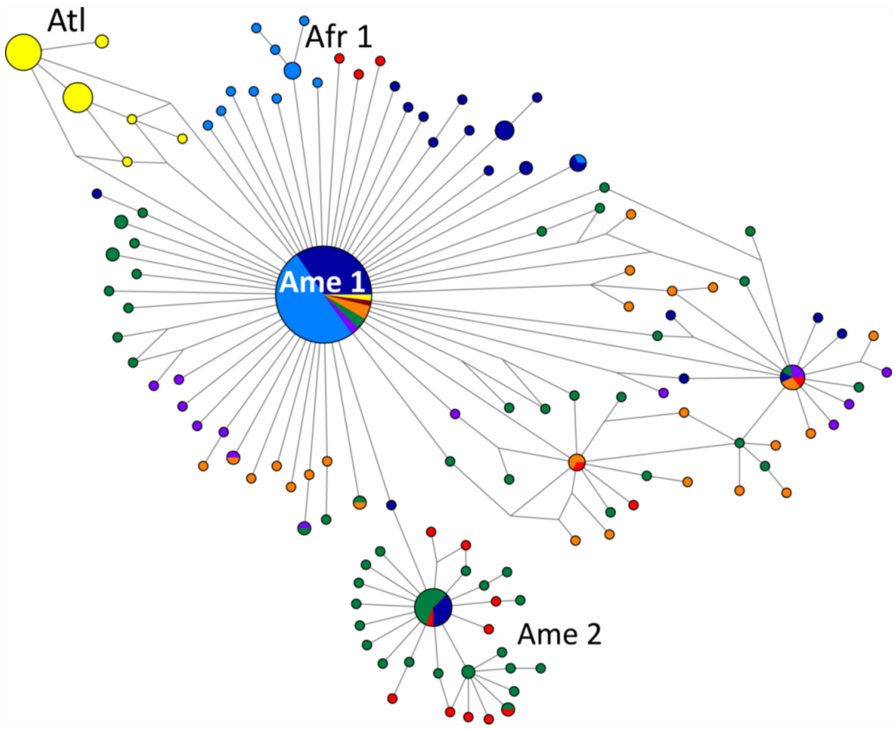

Supplementary Fig. 1. Median-joining network of *Plasmodium vivax/P. simium* mitochondrial lineages from South and Central America. Circle sizes are proportional to haplotype frequencies, and pairs of haplotypes connected by a straight line differ by a single mutational step. Clades Ame 1, Ame2, Atl, and Afr1 are identified in the network. The following color code was used to indicate the geographic origin of isolates: dark blue = Brazil; light blue = Central America and Mexico; orange = Peru; green = Colombia; dark red (wine) = Ecuador; yellow = Atlantic Forest of Southeast and South Brazil, and red = Venezuela.

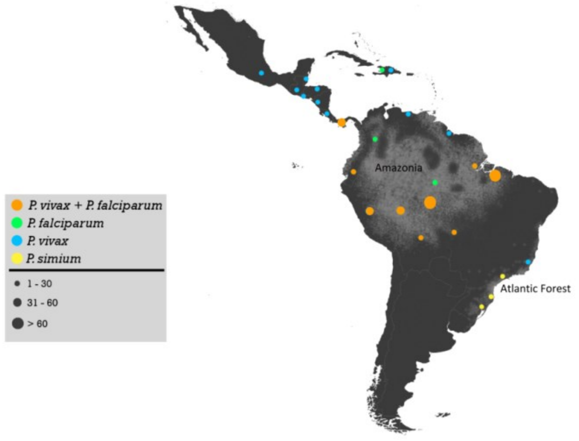

Supplementary Fig. 2. Map showing the collection sites of the New World samples of *Plasmodium falciparum* and *P. vivax*/*P. simium* analyzed in this study. The map was built using the open-access R software library *rworldmap: mapping global data* combined with the *ggplot2* library, which are both available at http://www.R-project.org/.

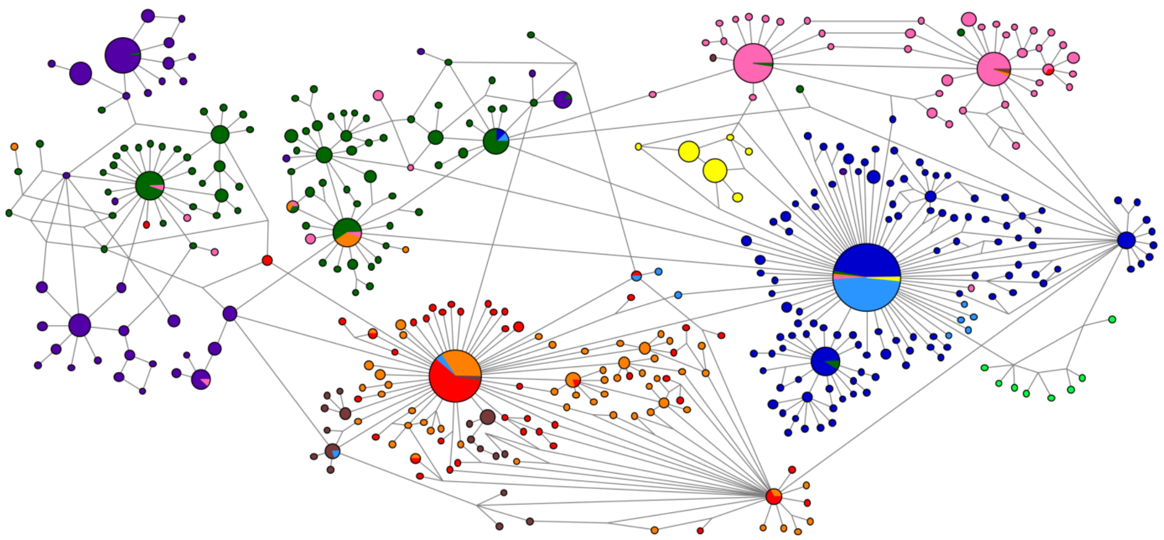

Supplemetary Fig. 3. Median-joining network of the global sample of *Plasmodium vivax* mitochondrial lineages, including *P. vivax*-like parasites from African great apes. As in Fig. 2b, circle sizes are proportional to haplotype frequencies, and pairs of haplotypes connected by a straight line differ by a single mutational step. The following color code was used to identify the geographic origin of parasites: red = Africa (human samples), light green = African great apes (data from[27]), dark blue = South America, light blue = Central America and Mexico, yellow = Atlantic Forest from southeast and South Brazil, brown = Middle East and Central Asia, orange = South Asia, green = Southeast Asia, dark purple = East Asia, and pink = Melanesia.

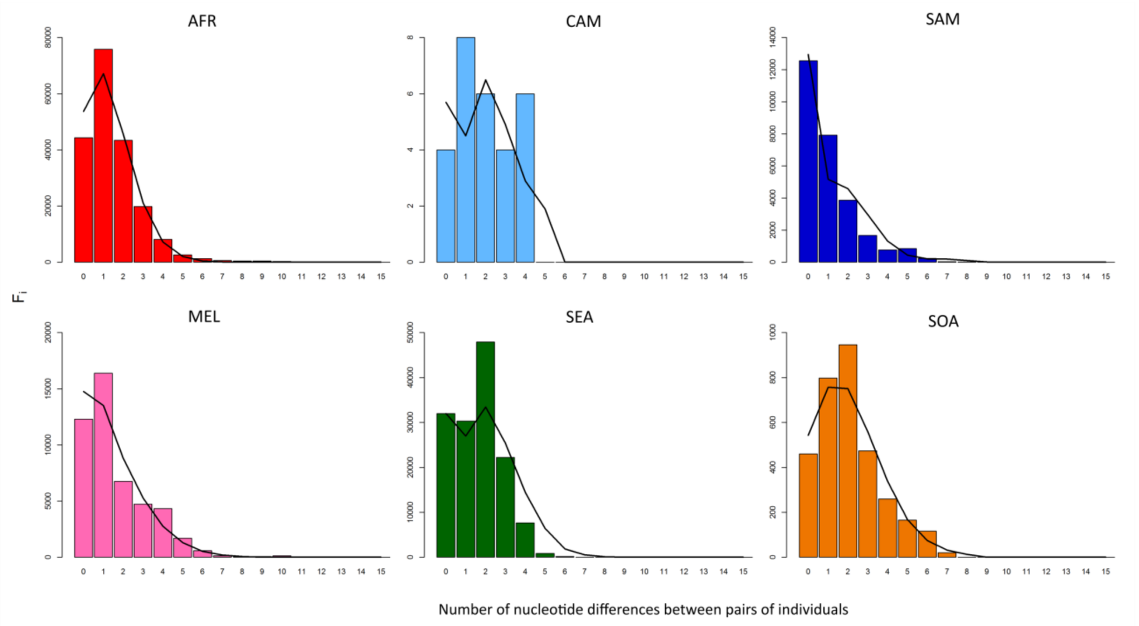

Supplementary Fig. 4 Mismatch distribution analysis of mitochondrial lineages from regional *Plasmodium falciparum* populations. The following populations were analyzed: Africa (AFR; red), South America (SAM; dark blue), Central America (CAM; light blue), South Asia (SOA; orange), Southeast Asia (SEA; green), and Melanesia (MEL; pink). Bars show observed frequencies, and continuous lines show expected frequencies under a sudden population expansion model[76]. Note that observed and expected distributions are quite similar in most populations, except for CAM (n = 8 isolates) (see also S11 for significance tests).

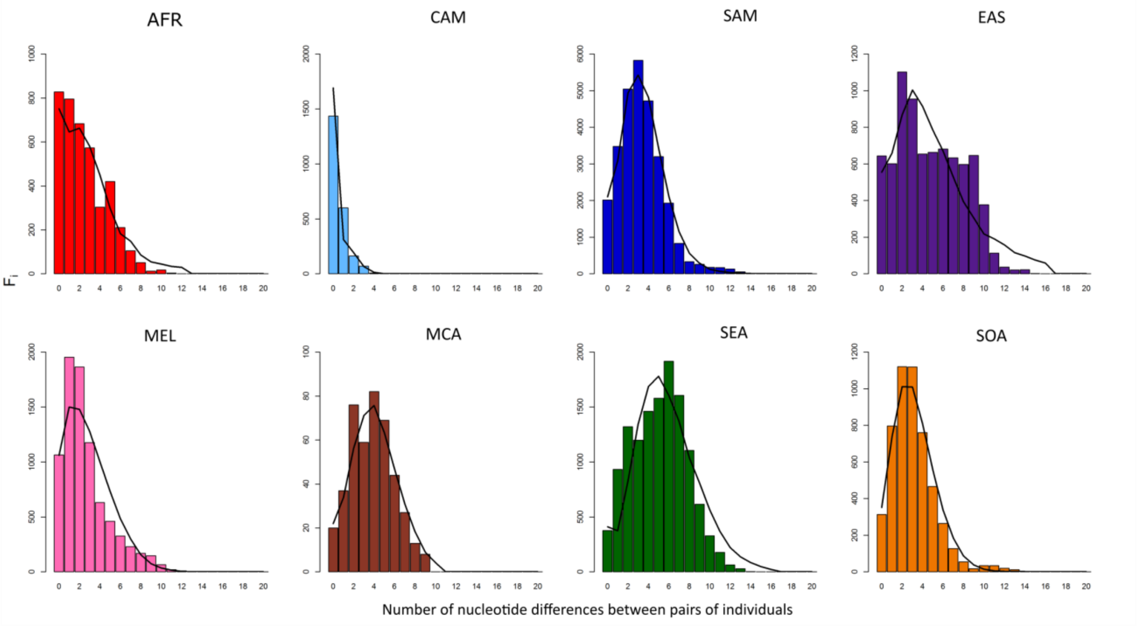

Supplementary Fig. 5. Mismatch distribution analysis of mitochondrial lineages from regional *Plasmodium vivax* populations. The following populations were analyzed: Africa (AFR; red), South America (SAM; dark blue), Central America and Mexico (CAM; light blue), Middle East and Central Asia combined (MCA; brown), South Asia (SOA; orange), Southeast Asia (SEA; green), East Asia (EAS; dark purple), and Melanesia (MEL; pink). Bars show observed frequencies, and continuous lines show expected frequencies under a sudden population expansion model[76]. Note that observed and expected distributions are quite similar in all populations (see also Supplementary Table 11 for significance tests).

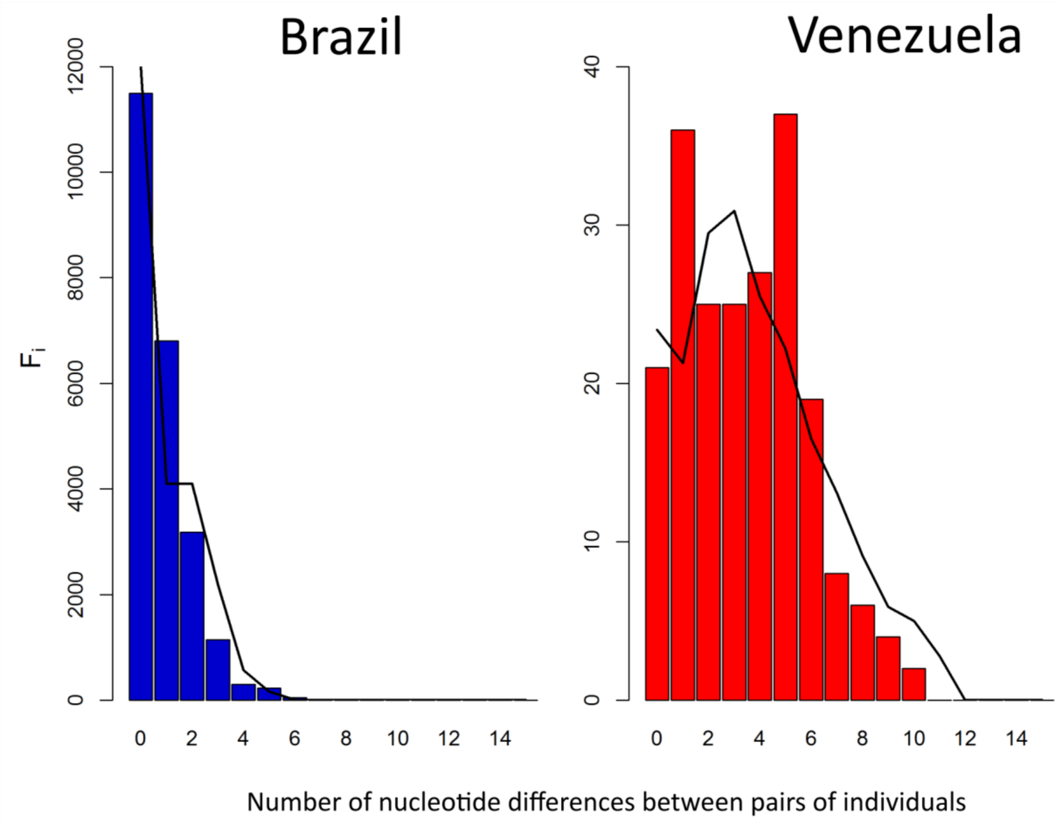

Supplementary Fig. 6. Country-specific mismatch distribution analyses of mitochondrial lineages from *Plasmodium falciparum* populations from South America. Bars show observed frequencies, and continuous lines show expected frequencies under a sudden population expansion model[76]. See Supplementary Table 12 for significance tests.

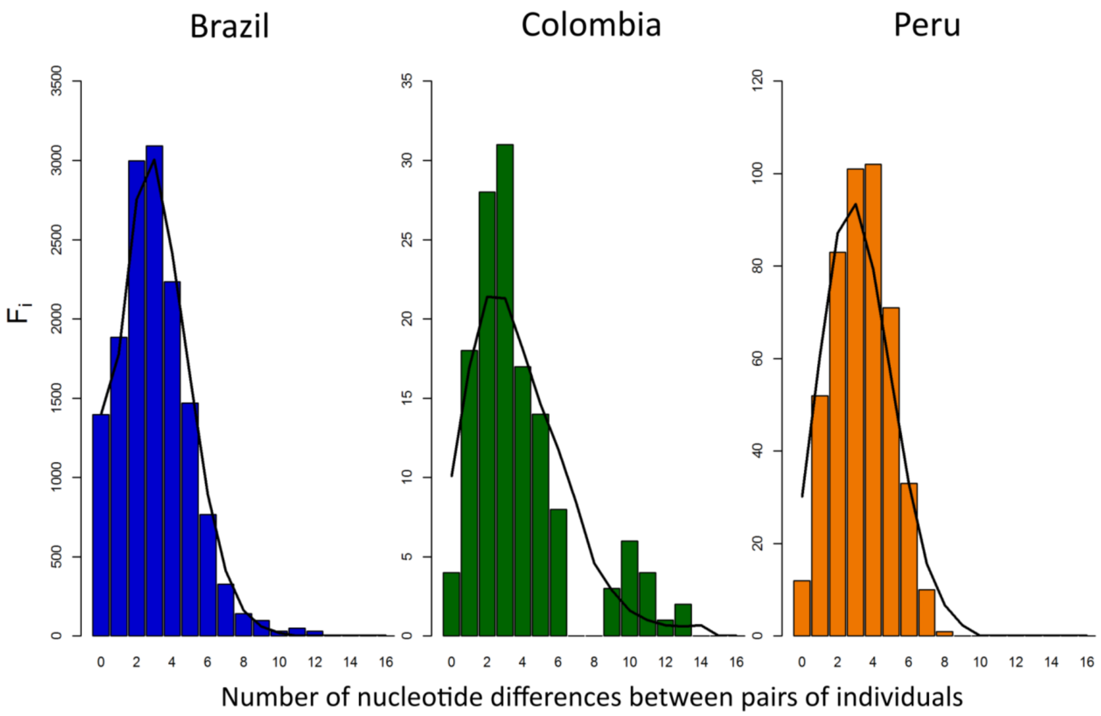

Supplementary Fig. 7. Country-specific mismatch distribution analyses of mitochondrial lineages from *Plasmodium vivax* populations from South America. Bars show observed frequencies, and continuous lines show expected frequencies under a sudden population expansion model[76]. See Supplementary Table 12 for significance tests.

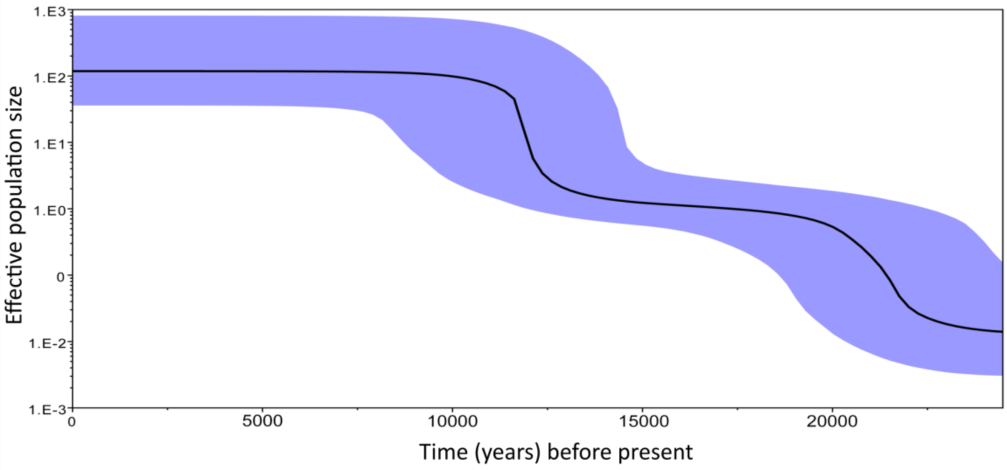

Supplementary Fig. 8. Bayesian skyline plot of the global population of *Plasmodium falciparum* showing changes in effective population size N_e (shown in log scale on y-axis) over time (x-axis). The black line shows the median ancestral population size, whereas the colored blue region shows the 95% highest probability density (HPD) interval for this estimate.

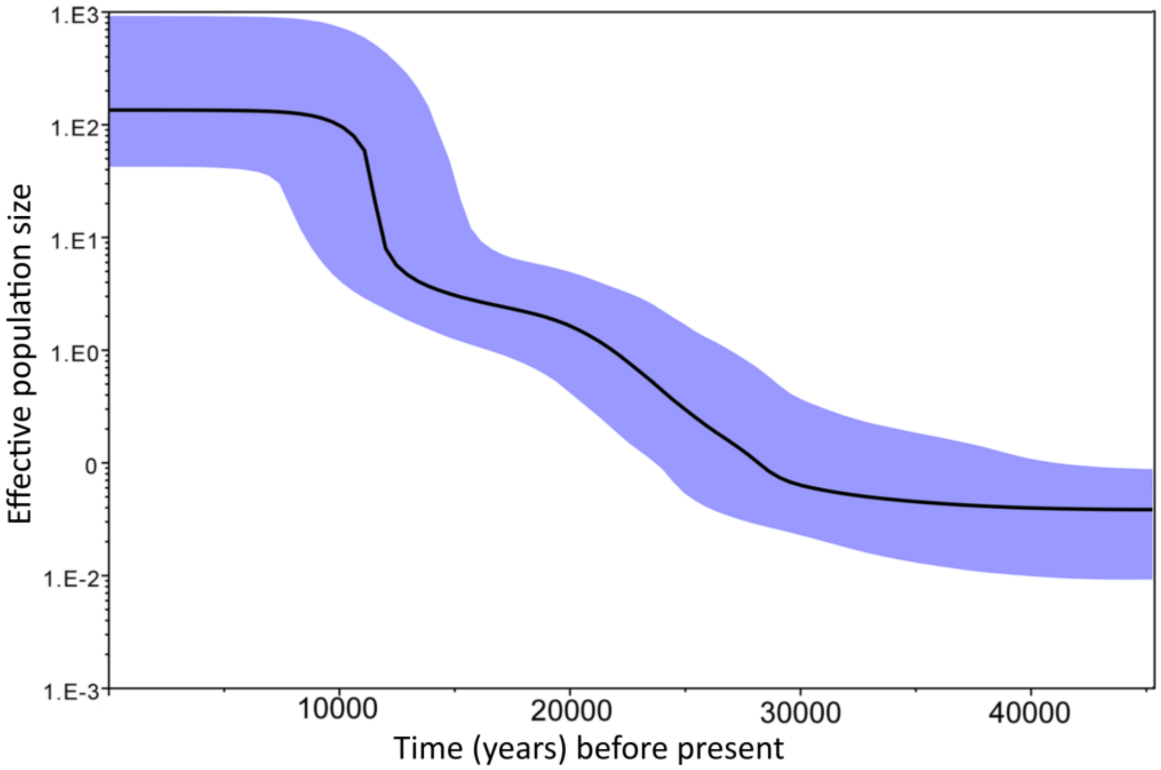

Supplementary Fig. 9. Bayesian skyline plot of the global population of *Plasmodium vivax* showing changes in effective population size N_e (shown in log scale on y-axis) over time (x-axis). The black line shows the median ancestral population size, whereas the colored blue region shows the 95% highest probability density (HPD) interval for this estimate.

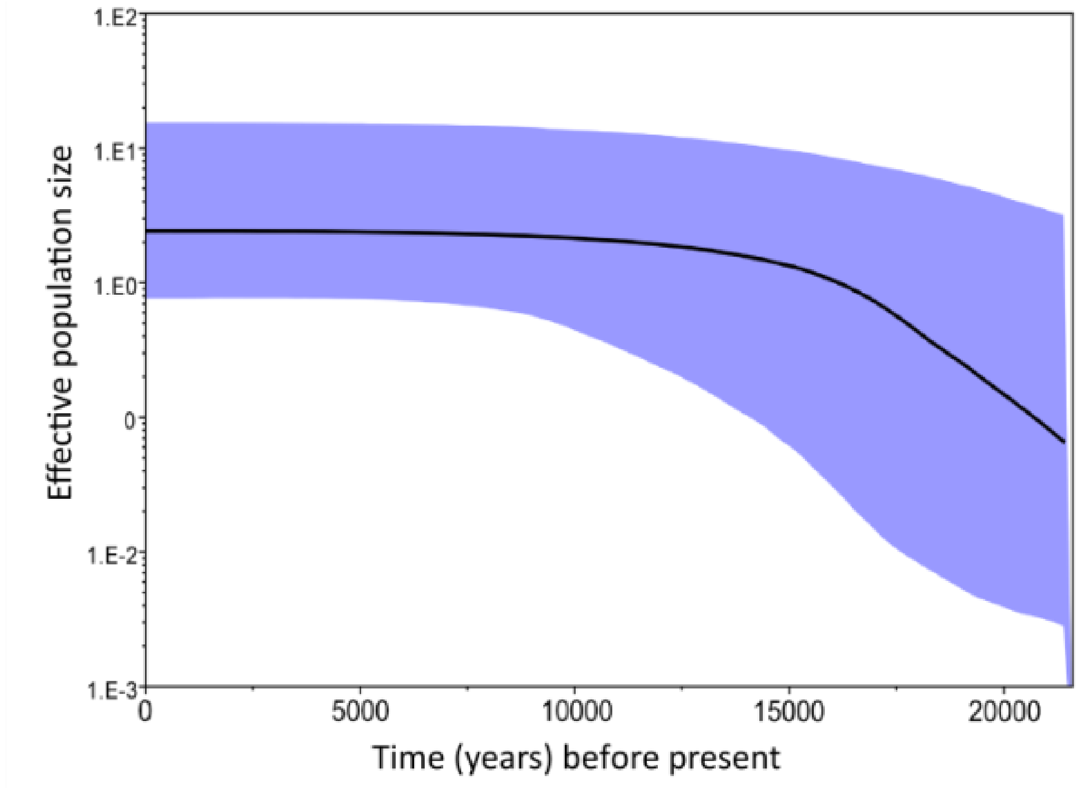

Supplementary Fig. 10. Bayesian skyline plot of the SAM population of Plasmodium falciparum showing changes in effective population size Ne (shown in log scale on y-axis) over time (x-axis). The black line shows the median ancestral population size, whereas the colored blue region shows the 95% highest probability density (HPD) interval for this estimate.

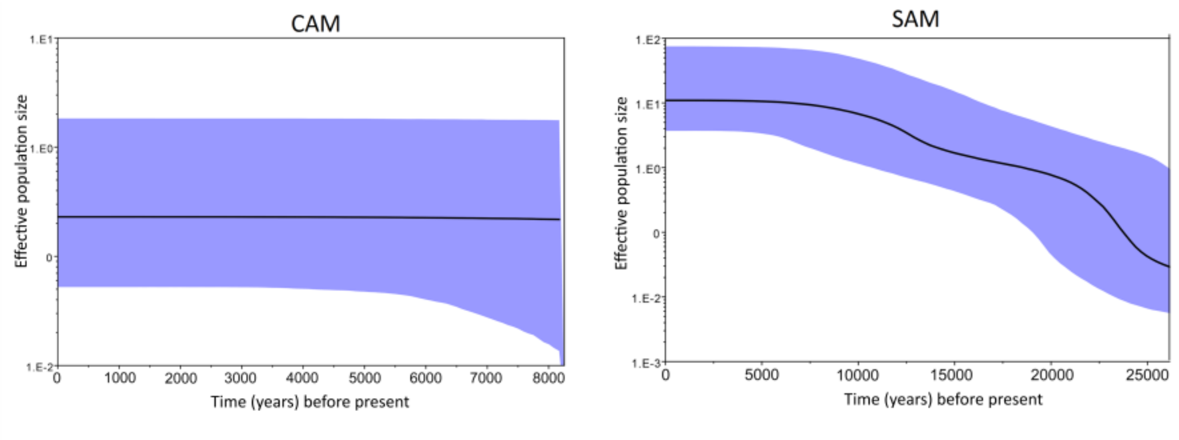

Supplementary Fig. 11. Bayesian skyline plot of the SAM and CAM populations of *Plasmodium vivax* showing changes in effective population size N_e (shown in log scale on y-axis) over time (x-axis). The black line shows the median ancestral population size, whereas the colored blue region shows the 95% highest probability density (HPD) interval for this estimate. SAM = South America; CAM = Central America and Mexico.

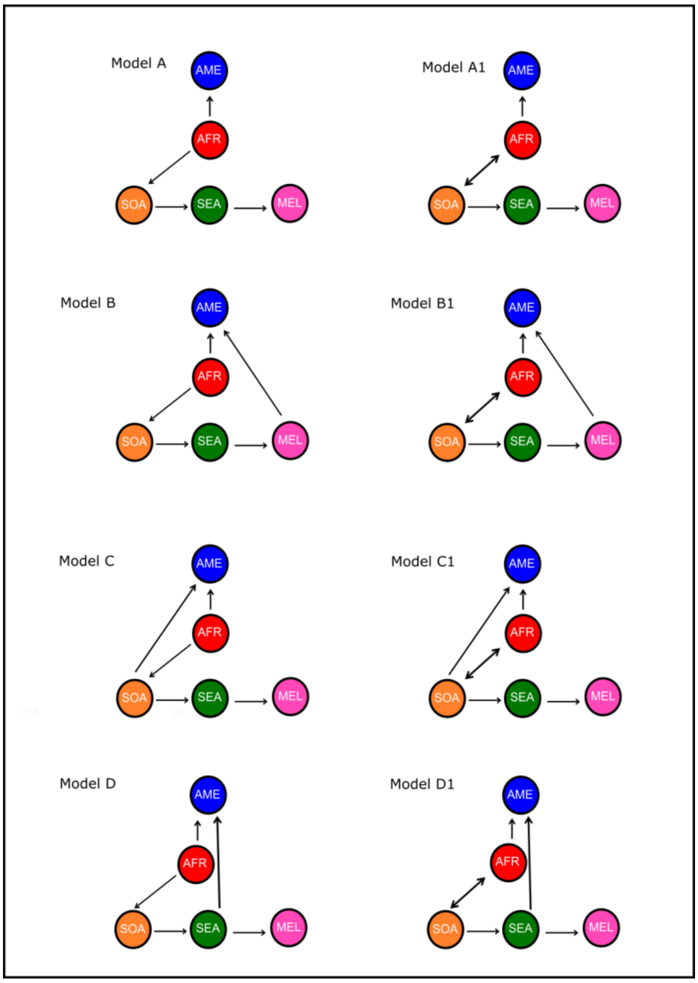

Supplementary Fig. 12. A priori *Plasmodium falciparum* migration models compared using Migrate-N to make inferences regarding gene flow between regional populations. Colored circles represent the following regional populations: Africa (AFR; red), South and Central America combined (SAM; dark blue), South Asia (SOA; orange), Southeast Asia (SEA; green), and Melanesia (MEL; pink).

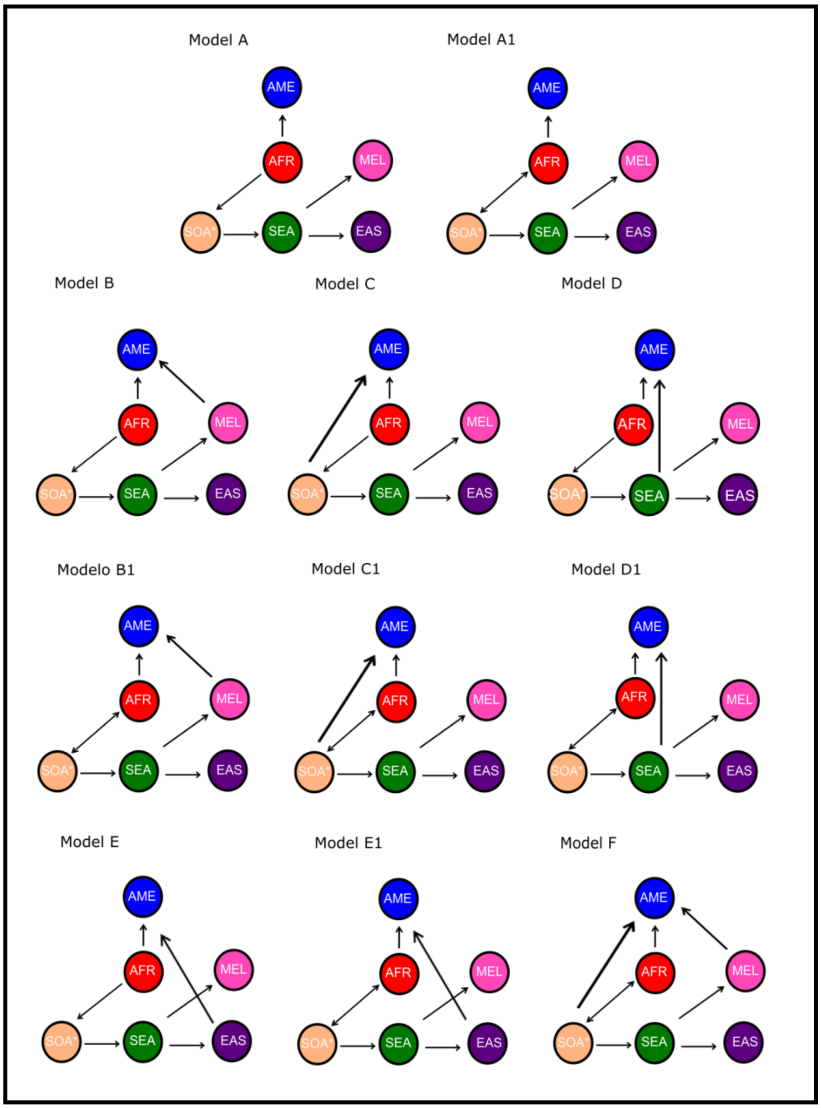

Supplementary Fig. 13. A priori migration models assuming an African origin of *Plasmodium vivax* that were compared using Migrate-N to make inferences regarding gene flow between regional populations. Colored circles represent the following populations: Africa (AFR; red), South and Central America (SAM; dark blue), Middle East, Central and South Asia combined (SOA*; light brown), Southeast Asia (SEA; green), East Asia (EAS; dark purple), and Melanesia (MEL; pink).

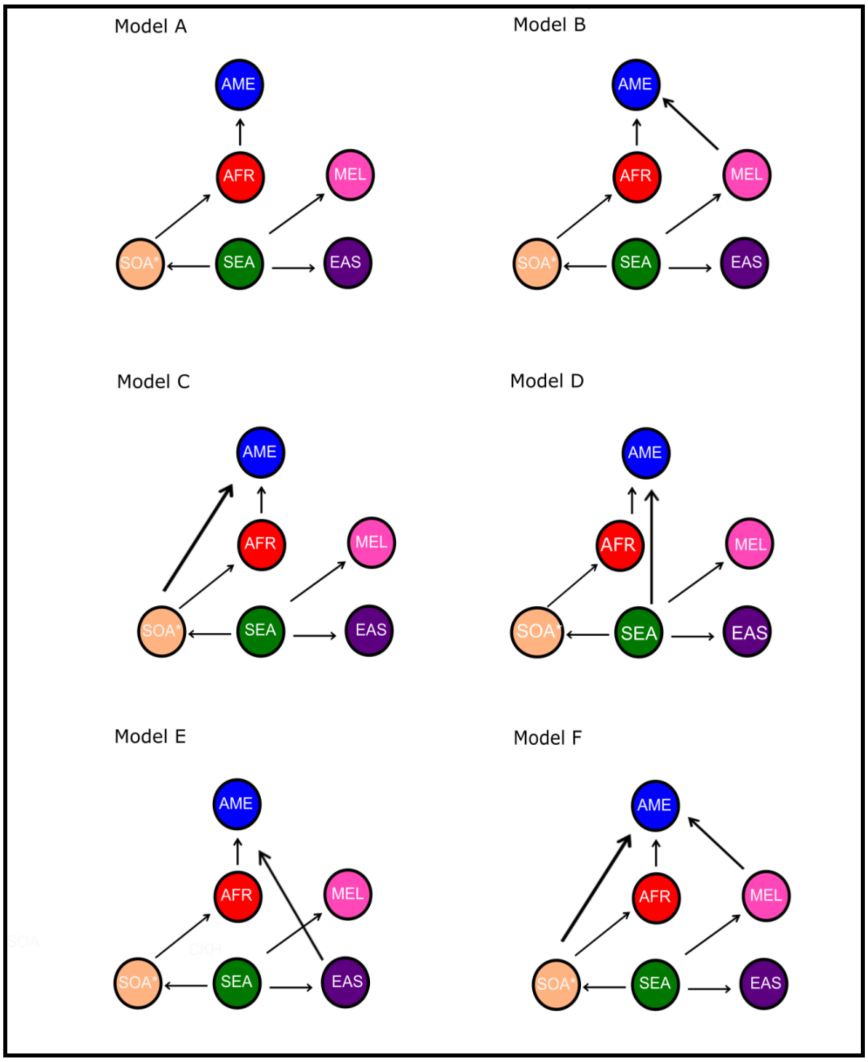

Supplementary Fig. 14. A priori migration models assuming a Southeast Asian origin of *Plasmodium vivax* that were compared using Migrate-N to make inferences regarding gene flow between regional populations. Colored circles represent the following populations: Africa (AFR; red), South and Central America (SAM; dark blue), Middle East, Central and South Asia combined (SOA*; light brown), Southeast Asia (SEA; green), East Asia (EAS; dark purple), and Melanesia (MEL; pink).

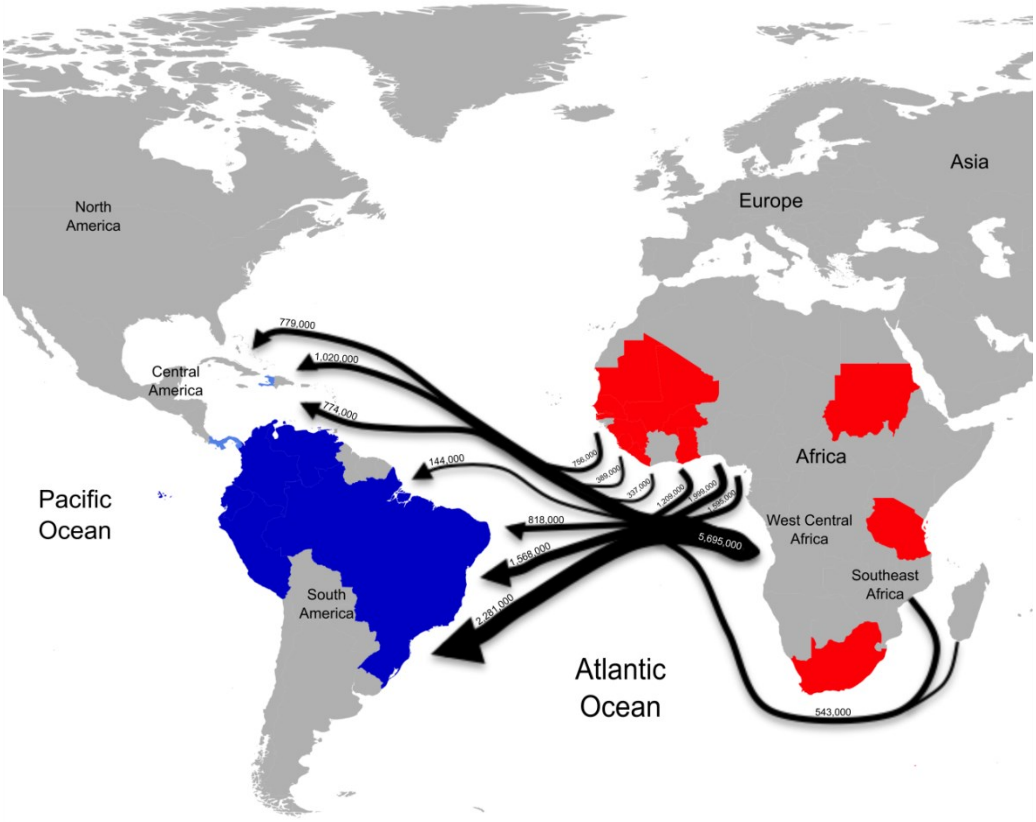

Supplementary Fig. 15. Map showing the geographic origins of enslaved Africans brought to the Americas between the 1500s and the mid-1800s. Figures next to arrows represent estimates of total numbers of slaves according to each route, which were derived from the Trans-Atlantic Slave Trade Database of Emory University (http://www.slavevoyages.org[35]). The geographic origins of African and American mitochondrial lineages of *Plasmodium falciparum* analyzed in this study are indicated in the map at the country level using the same color code as that of Fig. 4a. Map built using the open-access R software library *rworldmap: mapping global data* combined with the *ggplot2* library, which are both available at http://www.R-project.org/.

Supplementary Tables

Supplementary Table 1. Country-specific levels of genetic diversity in *Plasmodium falciparum* mitogenomes from South America.

**Country**	**No. isolates**	**Nucleotide diversity**		**H^{d} (SD)^{b}**
		**π^{a} (SD)^{b}**	**θs^{c} (SD)^{b}**	
Venezuela	21	0.00058 (0.00011)	0.00135 (0.00051)	0.900 (0.062)
Brazil	208	0.00014 (0.00002)	0.00079 (0.00022)	0.507 (0.041)
**Total**	**229**	**0.00018 (0.00002)**	**0.00153 (0.00038)**	**0.559 (0.039)**

$^{a}\pi$ = average number of pairwise nucleotide differences per site; ^{b}SD = standard deviation; $^{c}\theta_S$ = standardized number of segregating sites; and ^{d}H = haplotype diversity.

Supplementary Table 2. Country-specific levels of genetic diversity in *Plasmodium vivax* mitogenomes from South America.

Country	No. isolates	Nucleotide diversity		H^{d} (SD)
		π^{a} (SD)^{b}	θs^{c} (SD)^{c}	
Venezuela	15	0.00090 (0.00014)	0.00159 (0.00063)	1 (0.024)
Colombia	17	0.00066 (0.00015)	0.00148 (0.00058)	0.971 (0.032)
Peru	31	0.00058 (0.00006)	0.00151 (0.00052)	0.974 (0.020)
Brazil	171	0.00052 (0.00003)	0.00265 (0.00064)	0.904 (0.017)
**Total**	**234**	**0.00058 (0.00003)**	**0.00328 (0.00075)**	**0.9323 (0.019)**

$^{a}\pi$ = average number of pairwise nucleotide differences per site; ^{b}SD = standard deviation; $^{c}\theta_S$ = standardized number of segregating sites; and ^{d}H = haplotype diversity.

Supplementary Table 3. Number of *Plasmodium falciparum* mitogenome haplotypes that are unique within regional populations and shared between populations.

Regional population	Haplotypes			No. shared between populations					
	total	unique	shared	AFR[a]	SAM[b]	CAM[c]	SOA[d]	SEA[e]	MEL[f]
AFR[a]	191	179	12	-	4	2	5	7	5
SAM[b]	41	36	5		-	2	2	3	2
CAM[c]	5	2	3			-	1	1	1
SOA[d]	23	18	5				-	4	2
SEA[e]	56	48	8					-	4
MEL[f]	35	28	6						-

[a]AFR = Africa; [b]SAM = South America; [c]CAM = Central America; [d]SOA = South Asia; [e]SEA = Southeast Asia; and [f]MEL = Melanesia.

Supplementary Table 4. Pairwise genetic differentiation between mitogenomes of regional *Plasmodium falciparum* populations as estimated by Wright's fixation index F_{ST}. All F_{ST} values are significantly different from zero at the level of 0.05.

**Population**	**AFR[a]**	**SAM[b]**	**CAM[c]**	**SOA[d]**	**SEA[e]**	**MEL[f]**
AFR[a]	-					
SAM[b]	0.468	-				
CAM[c]	0.293	0.669	-			
SOA[d]	0.091	0.537	0.234	-		
SEA[e]	0.270	0.596	0.382	0.266	-	
MEL[f]	0.130	0.558	0.292	0.147	0.097	-

[a]AFR = Africa; [b]SAM = South America; [c]CAM = Central America; [d]SOA = South Asia; [e]SEA = Southeast Asia; and [f]MEL = Melanesia.

Supplementary Table 5. Number of *Plasmodium vivax* mitogenome haplotypes that are unique within regional populations and shared between populations.

Population	Haplotypes			No. shared between populations							
	total	unique	shared	AFR[a]	SAM[b]	CAM[c]	SOA[d]	SEA[e]	MEL[f]	MCA[g]	EAS[h]
AFR[a]	45	39	6	-	0	2	5	0	1	1	0
SAM[b]	119	116	3		-	2	1	2	1	0	0
CAM[c]	12	7	5			-	2	1	1	2	0
SOA[d]	62	54	8				-	1	3	2	0
SEA[e]	87	80	7					-	4	0	2
MEL[f]	51	43	8						-	1	1
MCA[g]	19	16	3							-	0
EAS[h]	37	34	3								-

[a]AFR = Africa; [b]SAM = South America; [c]CAM = Central America and Mexico; [d]SOA = South Asia; [e]SEA = Southeast Asia; [f]MEL = Melanesia; [g]MCA = Middle East and Central Asia; and [h]EAS = East Asia.

Supplementary Table 6. Pairwise genetic differentiation between mitogenomes of regional *Plasmodium vivax* populations, as estimated by Wright's fixation index $F_{ST.}$ All F_{ST} values are significantly different from zero at the level of 0.05.

Population	AFR[a]	SAM[b]	CAM[c]	SOA[d]	SEA[e]	MEL[f]	MCA[g]	EAS[h]
AFR[a]	-							
SAM[b]	0.262	-						
CAM[c]	0.343	0.050	-					
SOA[d]	0.014	0.228	0.260	-				
SEA[e]	0.241	0.245	0.228	0.211	-			
MEL[f]	0.406	0.242	0.345	0.360	0.320	-		
MCA[g]	0.087	0.269	0.400	0.075	0.212	0.389	-	
EAS[h]	0.345	0.457	0.414	0.343	0.203	0.470	0.305	-

[a]AFR = Africa; [b]SAM = South America; [c]CAM = Central America and Mexico; [d]SOA = South Asia; [e]SEA = Southeast Asia; [f]MEL = Melanesia; [g]MCA = Middle East and Central Asia; and [h]EAS = East Asia.

Supplementary Table 7. Place and date of collection and original host of 32 *P. vivax*/*P. simium* isolates from the Atlantic Forest of South and Southeast Brazil.

Isolate code	Place (year) of collection	Host	Haplotype	Reference
Fonseca MRA-353	Cantareira State Park, São Paulo (1960s)	Howler monkey (*Alouatta clamitans*)	*Atl2*	Jongwutiwes *et al.*[1]
SS3Ps	Cantareira State Park, São Paulo (2007)	Howler monkey (*Alouatta clamitans*)	*Atl1*	Duarte *et al.*[2]
139Ps	Parelheiros, São Paulo (2003)	Howler monkey (*Alouatta clamitans*)	*Atl1*	Duarte *et al.*[2]
BL65Ps	Indaial, Santa Catarina (2011)	Howler monkey (*Alouatta clamitans*)	*Atl1*	Costa *et al.*[3]
RSPs	Itapuã State Park, Rio Grande do Sul (2003)	Howler monkey (*Alouatta clamitans*)	*Atl1*	Duarte *et al*. unpublished
160Ps	Cantareira State Park, São Paulo (2010)	Black-fronted titi monkey (*Callicebus nigrifrons*)	*Atl1*	Bueno[4]
121Ps	Cantareira State Park, São Paulo (2003)	Howler monkey (*Alouatta clamitans*)	*Atl1*	Duarte *et al.*[2]
95Ps	Cantareira State Park, São Paulo (2009)	Howler monkey (*Alouatta clamitans*)	*Atl1*	Yamasaki *et al.*[5]
97Ps	Mariporã, São Paulo (2009)	Howler monkey (*Alouatta clamitans*)	*Atl1*	Yamasaki *et al.*[5]
45Ps	Parelheiros, São Paulo (2007)	Howler monkey (*Alouatta clamitans*)	*Atl1*	Duarte *et al.*[2]
1312MT	Santa Maria de Jetibá, Espírito Santo (2003)	Human	*Atl1*	Cerutti *et al.*[6]
JSB62MT	Santa Teresa, Espírito Santo (2002)	Human	other	Cerutti *et al.*[6]
1565MT	Santa Leopoldina, Espírito Santo (2003)	Human	*Atl1*	Cerutti *et al.*[6]
GAB847MT	Santa Maria de Jetibá, Espírito Santo (2002)	Human	*Atl2*	Cerutti *et al.*[6]
RO54MT	Domingos Martins, Espírito Santo (2002)	Human	other	Cerutti *et al.*[6]
VC57MT	Santa Teresa, Espírito Santo (2002)	Human	*Atl1*	Cerutti *et al.*[6]
1272MT	Alfredo Chaves, Espírito Santo (2003)	Human	*Atl2*	Cerutti *et al.*[6]
OJA51MT	Santa Teresa, Espírito Santo (2002)	Human	*Atl1*	Cerutti *et al.*[6]
1411MT	Santa Leopoldina, Espírito Santo (2003)	Human	*Atl2*	Cerutti *et al.*[6]
1760MT	Espírito Santo	Human	*Atl2*	Cerutti *et al.*[6]
1451MT	Santa Teresa, Espírito Santo (2003)	Human	*Atl2*	Cerutti *et al.*[6]
ACC54MT	Santa Teresa, Espírito Santo (2002)	Human	*Atl1*	Cerutti *et al.*[6]
FW63MT	Santa Teresa, Espírito Santo (2002)	Human	*Atl2*	Cerutti *et al.*[6]
MA5M61MT	Espírito Santo	Human	other	Cerutti *et al.*[6]
SV555MT	Marechal Floriano, Espírito Santo (2002)	Human	*Atl1*	Cerutti *et al.*[6]
143MT	Santa Teresa, Espírito Santo (2002)	Human	*Atl2*	Cerutti *et al.*[6]

40MT	Santa Teresa, Espírito Santo (2002)	Human	*Ame1*	Cerutti *et al*.[6]
103_03MT	Espírito Santo	Human	*Ame1*	Cerutti *et al*.[6]
111MT	Santa Teresa, Espírito Santo (2002)	Human	*Atl2*	Cerutti *et al*.[6]
761MT	Santa Leopoldina, Espírito Santo (2002)	Human	other	Cerutti *et al*.[6]
ALNL53MT	Santa Teresa, Espírito Santo (2002)	Human	other	Cerutti *et al*.[6]
AJ54MT	Santa Teresa, Espírito Santo (2002)	Human	*Atl2*	Cerutti *et al*.[6]

Supplementary Table 8. Private single-nucleotide polymorphisms (indicated by boldface letters) in the mitogenome of 32 *P. vivax*/*P. simium* isolates from the Atlantic Forest of South and Southeast Brazil.

**Isolate code**	**Nucleotide position (gene)**				
	**C1342G**	**A3325T**	**T4134C**	**A4468G**	**A5322C**
	Intergenic	*cox1*	*cox1*	*cox1*	*cytb*
Fonseca MRA-353	C	**T**	**C**	**G**	A
SS3Ps	C	A	**C**	**G**	A
139Ps	C	A	**C**	**G**	A
Bl65Ps	C	A	**C**	**G**	A
R5Ps	C	A	**C**	**G**	A
160Ps	C	A	**C**	**G**	A
121Ps	C	A	**C**	**G**	A
95Ps	C	A	**C**	**G**	A
97Ps	C	A	**C**	**G**	A
45Ps	C	A	**C**	**G**	A
1312MT	C	A	**C**	**G**	A
JSB62_MT	**G**	A	**C**	**G**	A
1565MT	C	A	**C**	**G**	A
GAB847_MT	C	**T**	**C**	**G**	A
RO54_MT	**G**	A	**C**	**G**	A
VC57MT	C	A	**C**	**G**	A
1272MT	C	**T**	**C**	**G**	A
OJA51_MT	C	A	**C**	**G**	A
1411MT	C	**T**	**C**	**G**	A
1760MT	C	**T**	**C**	**G**	A
1451MT	C	**T**	**C**	**G**	A
ACC54_MT	C	A	**C**	**G**	A
FW63MT	C	**T**	**C**	**G**	A
MA5M61_MT	C	**T**	T	**G**	A
SV555_MT	C	A	**C**	**G**	A
143MT	C	**T**	**C**	**G**	A
40MT	C	A	T	A	A
103_03MT	C	A	T	A	A
111MT	C	**T**	**C**	**G**	A
761MT	C	**T**	**C**	A	A
ALNL53MT	C	**T**	T	**G**	**C**
AJR54_MT	C	**T**	**C**	**G**	A

Supplementary Table 9. The results of Tajima's *D* and Fu's F_S neutrality tests applied to *Plasmodium falciparum* mitogenomes from South America. Statistically significant *P* values are underlined.

Country	Neutrality test			
	**Tajima's D**	***P value***	**Fu's F_s**	***P value***
Venezuela	-2.18761	0.006	-3.2007	<0.02
Brazil	-2.34520	<0.0001	-5.05091	<0.02
**Total**	**-2.61249**	**0.001**	**-8.47147**	**<0.02**

Supplementary Table 10. The results of Tajima's *D* and Fu's F_s neutrality tests applied to *Plasmodium vivax* mitogenomes from South America. Statistically significant *P* values are underlined.

**Country**	**Neutrality test**			
	**Tajima's *D***	***P* value**	**Fu's F_s**	***P* value**
Venezuela	-1.84870	0.01	-2.46987	<0.05
Colombia	-2.26611	<0.0001	-3.19186	<0.02
Peru	-4.76892	<0.0001	-3.90115	<0.02
Brazil	-2.50856	<0.0001	-6.25391	<0.02
**Total**	-2.52771	0.001	0.16976	>0.10

Supplementary Table 11. Sum of square deviations (SDD) and raggedness index (R) comparing the observed mismatch distribution in each of the regional populations of *Plasmodium falciparum* and *P. vivax* with the expected distribution under a sudden demographic expansion model; *P* values > 0.05 indicate an expanding population.

Population	*P. falciparum*		*P. vivax*	
	SSD (*P* value)	R (*P* value)	SSD (*P* value)	R (*P* value)
AFR[a]	0.00267 (<0.001)	0.07110 (<0.001)	0.00237 (0.88)	0.01068 (0.97)
SAM[b]	0.00043 (0.80)	0.05627 (0.81)	0.00024 (0.95)	0.01488 (0.80)
EAS[c]	-	-	0.00583 (0.64)	0.00866 (0.89)
MCA[d]	-	-	0.00357 (0.43)	0.02112 (0.52)
MEL[e]	0.00916 (0.16)	0.05466 (0.26)	0.00124 (0.68)	0.02483 (0.43)
CAM[f]	0.01577 (0.44)	0.08163 (0.56)	0.00023 (0.52)	0.17341 (0.79)
SEA[g]	0.01135 (0.22)	0.06169 (0.28)	0.00594 (0.17)	0.00852 (0.75)
SOA[h]	0.00287 (0.12)	0.04055 (0.03)	0.00023 (0.78)	0.02336 (0.38)

[a]AFR = Africa; [b]SAM = South America; [c]EAS = East Asia (only *P. vivax*); [d]MCA = Middle East and Central Asia (only *P. vivax*); [e]MEL = Melanesia; [f]CAM = Central America and Mexico; [g]SEA = Southeast Asia; and [h]SOA = South Asia.

Supplementary Table 12. Sum of square deviations (SDD) and raggedness index (R) comparing the observed mismatch distribution for South American populations of *Plasmodium falciparum* and *P. vivax* with the expected distribution under a sudden demographic expansion model; *P* values > 0.05 indicate an expanding population.

Country	*P. falciparum*		*P. vivax*	
	SSD (*P* value)	R (*P* value)	SSD (*P* value)	R (*P* value)
Brazil	0.00005 (0.88)	0.07416 (0.76)	0.00013 (1.00)	0.01671 (0.87)
Colombia	-	-	0.00894 (0.45)	0.03492 (0.43)
Peru	-	-	0.00362 (0.09)	0.02729 (0.26)
Venezuela	0.01168 (0.58)	0.02066 (0.92)	-	-

Supplementary Table 13. Comparison of *Plasmodium falciparum* migration models estimated by Migrate-N; models are described in Supplementary Fig. 12.

Model	No. parameters[a]	Bézier log mL[b]	LBF[c]	Model probability
A	4 M, 5 Θ	-14736.87	-93.19	<0.00000001
B	5 M, 5 Θ	-14782.59	-138.91	<0.00000001
C	5 M, 5 Θ	-14732,10	-88.42	<0.00000001
D	5 M, 5 Θ	-14740.11	-96.43	<0.00000001
A1	5 M, 5 Θ	-14644.82	-1.14	0.24030838
B1	6 M, 5 Θ	-14648.19	-4.51	0.00825825
C1	6 M, 5 Θ	-14643.68	0.00	0.75143332
D1	6 M, 5 Θ	-14660.09	-16.41	0.00000005

[a]Number of parameters estimated in the model: M = median mutation-scaled pairwise migration rate and Θ = median mutation-scaled effective population size; [b]Bézier log mL = Bézier approximation of log marginal likelihood; [c]LBF = log Bayes factor.

Supplementary Table 14. Comparison of migration models assuming an African origin of *Plasmodium vivax* estimated with Migrate-N; models are described in Supplementary Fig. 13.

Model	No. parameters[a]	Bézier log mL[b]	LBF[c]	Model probability
A	5 *M*, 6 Θ	-15984.80	-53.92	<0.0000001
B	6 *M*, 6 Θ	-15958.54	-27.66	<0.0000001
C	6 *M*, 6 Θ	-15969.08	-38.20	<0.0000001
D	6 *M*, 6 Θ	-15980.13	-49.25	<0.0000001
E	6 *M*, 6 Θ	-15957.98	-27.10	<0.0000001
A1	6 *M*, 6 Θ	-15933.73	-2.850	0.0533535
B1	7 *M*, 6 Θ	-15934.53	-3.65	0.0239896
C1	7 *M*, 6 Θ	-15938.62	-7.74	0.0003817
D1	7 *M*, 6 Θ	-16001.45	-70.57	<0.0000001
E1	7 *M*, 6 Θ	-15964.90	-34.02	<0.0000001
F	8 *M*, 6 Θ	-15930.88	0.00	0.9223650

[a]Number of parameters estimated in the model: *M* = median mutation-scaled pairwise migration rate and Θ = median mutation-scaled effective population size; [b]Bézier log mL = Bézier approximation of log marginal likelihood; [c]LBF = log Bayes factor.

Supplementary Table 15. Comparison of migration models assuming a Southeast Asian origin of *Plasmodium vivax* estimated with Migrate-N; models are described in Supplementary Fig. 14.

Model	No. parameters[a]	Bézier log mL[b]	LBF[c]	Model probability
A	5 M, 6 Θ	-15929,99811	-16.64	<0.00000001
B	6 M, 6 Θ	-15913,36034	0.00	0.99999500
C	6 M, 6 Θ	-15969,51376	-56.15	<0.00000001
D	6 M, 6 Θ	-15963,05640	-49.70	<0.00000001
E	6 M, 6 Θ	-15936,12640	-22.77	<0.00000001
F	7 M, 6 Θ	-15925,55080	-12.19	0.00000499

[a]Number of parameters estimated in the model: *M* = median mutation-scaled pairwise migration rate and Θ = median mutation-scaled effective population size; [b]Bézier log mL = Bézier approximation of log marginal likelihood; [c]LBF = log Bayes factor.

Supplementary Table 16. Oligonucleotide primers used to amplify and sequence the mitogenomes.

P. falciparum - amplification and sequencing		
**Primer name**	**Sequence (5' - 3')**	**Amplicon size**
PfmtDNA1amp	F:5'-CGGGCAGATGTCAGTAACTT-3'	2001 bp
	R:5'-AGGGCTTAAACCAACAACAT-3'	
PfmtDNA2amp	F:5'-AGAACTCCAGGCGTTAACGT-3'	2013 bp
	R:5'-CACCTACCACTCCATAATTCTC-3'	
PfmtDNA3amp	F:5'-CAGGTGTATTTTTAGCAAGTCG-3'	2268 bp
	R:5'-GGCGCTTCCATTTATAAGAA-3'	
PfmtDNA1seq	F:5'-CGGGCAGATGTCAGTAACTT-3'	505 bp
	R:5'-TGAGCTGGGTTAAGAACGTC-3'	
PfmtDNA2seq	F:5'-CAAACTGCCTCAAGACGTTC-3'	490 bp
	R:5'-TTGGCATTTTGTTGAAATAGTC-3'	
PfmtDNA3seq	F:5'-TTTGGCATTTTGTTGAAATAGTC-3'	495 bp
	R:5'-TTATTCTTTACATATTTTTGGGGTAT-3'	
PfmtDNA4seq	F:5'-CAGAATAAAAACTTTCTCGAATAGAA-3'	516 bp
	R:5'-AGGGCTTAAACCAACAACAT-3'	
PfmtDNA5seq	F:5'-AGAACTCCAGGCGTTAACCT-3'	510 bp
	R:5'-CATTTTCTTGTGCAATTATTCTT-3'	
PfmtDNA6seq	F:5'-ACGTACTGAATTATATTCTTCATCTTT-3'	496 bp
	R:5'-TGTTAGCAATAACATTCCTGATG-3'	
PfmtDNA7seq	F:5'-TTATTGCTAACACTACCGGTTT-3'	512 bp
	R:5'-ACATCAATGGCAGCATTACC-3'	
PfmtDNA8seq	F:5'-GGTAATGCTGCCATTGATGT-3'	530 bp
	R:5'-TGCATATGAAACATCTGGTG-3'	
PfmtDNA9seq	F:5'-CAGGTGTATTTTTAGCAAGTCG-3'	479 bp
	R:5'-CCCTAAAGGATTTGTGCTACC-3'	
PfmtDNA10seq	F:5'-TTCTTACATTTACATGGTAGCACAA-3'	450 bp
	R:5'-ATCTTGTGGTAATTGACATCCA-3'	
PfmtDNA11 seq	F:5'-TTGGATGTCAATTACCACAAGA-3'	495 bp
	R:5'-GCATGCAATACCGAACATTT-3'	
PfmtDNA12seq	F:5'-GTATTGCATGCCTGGTGTTT-3'	482 bp
	R:5'-AAAAATGGCTGCTGGAAGTA-3'	
PfmtDNA13seq	F:5'-TTCTACTTCCAGCAGCCATT-3'	486 bp
	R:5'-ATGGAGCACTGGATTGGATA-3'	

***P. vivax* - amplification and sequencing**		
**Primer**	**Sequence (5' - 3')**	**Size**
Pv mtDNA1amp	F:5'-CGCTGACTTCCTGGCTAC-3'	2911 bp
	R:5'-GTCAGGCGTTAAAAGCGTTC-3'	
Pv mtDNA2amp	F:5'-TTGTACACACCGCTCGTCAC-3'	3497 bp
	R:5'-CCGAACCTTGGACTCTTGAA-3'	
Pv mtDNA1seq	F:5´ACGCTGACTTCCTGGCTAAA-3´	540 bp
	R:5´GGATGAAACCTTCCTGATCG-3´	
Pv mtDNA2seq	F:5´CACGAGTCGATCAGGAAGGT-3´	536 bp
	R:5´GTGTTGGCTGGGCATTAGTC-3´	
Pv mtDNA3seq	F:5´GACTAATGCCCAGCCAACAC-3´	566 bp
	R:5´GCTATCAAATGGCGAGAAGG-3´	

Pv mtDNA4seq	F:5´CCTTCTCGCCATTTGATAGC-3´	524 bp
	R:5´GCATCATGTATGACAGCATGTTT-3´	
Pv mtDNA5seq	F:5´TGCTGTCATACATGATGCACTT-3´	554 bp
	R:5´CAAGGCAACAATACACGCTAA-3´	
Pv mtDNA6seq	F:5´AGCGTGTATTGTTGCCTTGT-3´	303 bp
	R:5´CATCCATGTCAGGCGTTAAA-3´	
Pv mtDNA7seq	F:5´TTGTACACACCGCTCGTCAC-3´	495 bp
	R:5´AACTACCAAATAAAAATGAAAACCA-3´	
Pv mtDNA8seq	F:5´CAAATTGCAATCATAAAACTTTAGGTC-3´	460 bp
	R:5´CTAGCAATACCAGATACTAAAAGACCA-3´	
Pv mtDNA9seq	F:5´TCATTGTTGGTCTTTTAGTATCTGG-3´	530 bp
	R:5´CCAATTAAATATTTTTGTTCCAGTAGG-3´	
Pv mtDNA10seq	F:5´CCTACTGGAACAAAAATATTTAATTGG-3´	557 bp
	R:5´TTTAATGGGCATGGGTAATTT-3´	
Pv mtDNA11seq	F:5´AAATTACCCATGCCCATTAAA-3´	562 bp
	R:5´CCCTAAAGGATTTGTGCTACC-3´	
Pv mtDNA12seq	F:5´TGGTAGCACAAATCCTTTAGGG-3´	548 bp
	R:5´AAATGTTTGCTTGGGAGCTG-3´	
Pv mtDNA13seq	F:5´ACAGCTCCCAAGCAAACATT-3´	539 bp
	R:5´GACCGAACCTTGGACTCTTG-3´	
	***P. simium* - amplification and sequencing**	
**Primer**	**Sequence (5' - 3')**	**Size**
Amp1Ps	F:5'-TCCACACTTCAATTCGTACTTCC-3´	1147 bp
	R:5'-GCAAACACTAGCGGTGGAAT-3´	
Nest1_1Ps	F:5'-ACCAAAATATAATCTCCTGTTCTAATG-3´	533 bp
	R:5'-CAAGTCACTGATAATTCCGATGA-3´	
Nest1_2Ps	F:5'-CGGGCAGATGTCAGTAACTTG-3´	635 bp
	R:5'-TTGTTTCATTCGATAGTAAACGCTAT-3´	
Amp2Ps	F:5'-GCTCACGCATCGCTTCTAAC-3´	1287 bp
	R:5'-GATGAGACGACATGGAGGTG-3´	
Nest2_1Ps	F:5'-TGGAACCTTGTTCAAGTTCAAAT-3´	665 bp
	R:5'-TGCATCACTTCAAACAACTGAA-3´	
Nest2_2Ps	F:5'-CCTGTAACACAATAAAATAATGTACCA-3´	602 bp
	R:5'-CCCGGCGAACCTTCTTAC-3´	
Amp3Ps	F:5'-GCCGGGGATAACAGGTTATAGT-3´	1221 bp
	R:5'-TCTCCAGCAAATGTAGGATCAA-3´	
Nest3_1Ps	F:5'-GCCGGGGATAACAGGTTATAGT-3´	588 bp
	R:5'-CCTAAAGTTTTATGATTGCAATTTGT-3´	
Nest3_2Ps	F:5'-CAAATTGCAATCATAAAACTTTAGGTC-3´	623 bp
	R:5'-TGATAATAACATTAAAACACCACCTG-3´	
Amp4Ps	F:5'-TGCATCTAAGATCTAAAGGTTTAACA-3´	1326 bp
	R:5'-TTTGTCCCCAAGGTAAAACG-3´	
Nest4_1Ps	F:5'-TGTTTTAACAGGTGGTGTTTTAATG-3´	627 bp
	R:5'-GAATTTTCACGTAAATGTTTACCAA-3´	
Nest4_2Ps	F:5'-AGGTGCAATTATTGGATTGTTTA-3´	674 bp
	R:5'-AAGGTAAAACGTAACCTATAAAAGCTG-3´	
Amp5Ps	F:5'-TGCTACAGGTGCATCTCTTGTATT-3´	1636 bp
	R:5'-CAAAATGGCTGCTGGAAGTA-3´	
Nest5_1Ps	F:5'-CGTTTTACCTTGGGGACAAA-3´	708 bp
	R:5'-TGAACAAGTGCAAATAATCCAC-3´	

Nest5_2Ps	F:5'-TTGGATGTCAGTTACCACAAGA-3´<br>R:5'-ACGCGAAAAACCCTAGAACA-3´	765 bp

Supplementary Text 1: 18S rRNA gene sequence types in Plasmodium vivax isolates from the Amazon Basin of Brazil (Rondônia and Acre) and Sri Lanka (Kataragama)

Li et al. (7) have suggested that a single gene conversion event in the gene coding for the S-type of the 18S rRNA can be used to distinguish Old World (wild-type) from New World (variant) lineages of P. vivax. However, unpublished sequence data deposited in the GenBank database in 2005 by F. E. Merino and H. A. del Portillo (then at the University of São Paulo, Brazil) show that both Old World and New World types of 18S rRNA gene sequences are found in the Amazon Basin of Brazil (Rondônia and Acre) and in Sri Lanka (Kataragama), thus indicating that neither variant sequences are limited to the New World nor wild-type sequences circulate exclusively in the Old World. These data are summarized below.

GenBank accession	Sample code	Country: site	18S sequence type
DQ162614	MalDB36	Brazil: Rondonia	New World
DQ162600	MalDB37	Brazil: Rondonia	New World
DQ162604	MalDB39	Brazil: Rondonia	New World
DQ162598	MalDB56	Brazil: Rondonia	New World
DQ162608	MalDB58	Brazil: Rondonia	New World
DQ162615	MalDB59	Brazil: Rondonia	New World
DQ162602	MalDB60	Brazil: Rondonia	New World
DQ162599	MalDB63	Brazil: Rondonia	New World
DQ162603	MalDB65	Brazil: Rondonia	New World
DQ162601	MalDB66	Brazil: Rondonia	New World
DQ162611	MalDB176	Brazil: Acre	New World
DQ162607	MalDB177	Brazil: Acre	New World
DQ162612	MalDB178	Brazil: Acre	New World
DQ162609	MalDB179	Brazil: Acre	New World
DQ162613	MalDB180	Brazil: Acre	New World
DQ162595	MalDB181	Brazil: Acre	New World
DQ162610	MalDB182	Brazil: Acre	New World
DQ162606	MalDB184	Sri Lanka: Kataragama	New World
DQ162596	MalDB185	Sri Lanka: Kataragama	New World
DQ162597	MalDB187	Sri Lanka: Kataragama	New World
DQ162605	MalDB186	Brazil: Acre	New World
DQ162619	MalDB183	Sri Lanka: Kataragama	Old World

DQ162620	MalDB54	Brazil: Rondonia	Old World
DQ162616	MalDB41	Brazil: Rondonia	Old World
DQ162617	MalDB57	Brazil: Rondonia	Old World
DQ162618	MalDB61	Brazil: Rondonia	Old World

Supplementary References

1. Jongwutiwes, S. *et al*. Mitochondrial genome sequences support ancient population expansion in *Plasmodium vivax*. *Mol. Biol. Evol.* **22,** 1733-1739 (2005).
2. Duarte, A. M. *et al.* Natural *Plasmodium* infections in Brazilian wild monkeys: reservoirs for human infections? *Acta Trop.* **107,** 179-185 (2008).
3. Costa, D. C. *et al. Plasmodium simium/Plasmodium vivax* infections in southern brown howler monkeys from the Atlantic Forest. *Mem. Inst. Oswaldo. Cruz.* **109,** 641-653 (2014).
4. Bueno, M. G. *Pesquisa de Leishmania spp. e Plasmodium spp. em Primatas Neotropicais Provenientes de Regiões de Mata Atlântica e Amazônia Iimpactadas Por Ações Antrópicas: Iinvestigação in situ e ex situ. Doctoral dissertation.* (Faculty of Veterinary Medicine of the University of São Paulo, 2012).
5. Yamasaki, T. *et al.* Detection of etiological agents of malaria in howler monkeys from Atlantic Forests, rescued in regions of Sao Paulo city, Brazil. *J. Med. Primatol.* **40,** 392-400 (2011).
6. Cerutti, C., Jr. *et al.* Epidemiologic aspects of the malaria transmission cycle in an area of very low incidence in Brazil. *Malar. J.* **6,** 33 (2007).
7. Li. J. *et al.* Geographic subdivision of the range of the malaria parasite Plasmodium vivax. *Emerg Infect Dis*. **7**, 35-42 (2001).
